# A Novel hAPP/htau Mouse Model of Alzheimer's Disease: Inclusion of APP With Tau Exacerbates Behavioral Deficits and Zinc Administration Heightens Tangle Pathology

**DOI:** 10.3389/fnagi.2018.00382

**Published:** 2018-11-22

**Authors:** Stephen L. P. Lippi, Meghann L. Smith, Jane M. Flinn

**Affiliations:** Psychology Department, George Mason University, Fairfax, VA, United States

**Keywords:** mouse models, amyloid, tau, Barnes Maze, Zinc, behavior, activities of daily living

## Abstract

The brains of those with Alzheimer's disease have amyloid and tau pathology; thus, mice modeling AD should have both markers. In this study, we characterize offspring from the cross of the J20 (hAPP) and rTg4510 (htau) strains (referred to as dual Tg). Behavior was assessed at both 3.5 and 7 months, and biochemical differences were assessed at 8 months. Additionally, mice were placed on zinc (Zn) water or standard lab water in order to determine the role of this essential biometal. Behavioral measures examined cognition, emotion, and aspects of daily living. Transgenic mice (dual Tg and htau) showed significant deficits in spatial memory in the Barnes Maze at both 3.5 and 7 months compared to controls. At 7 months, dual Tg mice performed significantly worse than htau mice (*p* < 0.01). Open field and elevated zero maze (EZM) data indicated that dual Tg and htau mice displayed behavioral disinhibition compared to control mice at both 3.5 and 7 months (*p* < 0.001). Transgenic mice showed significant deficits in activities of daily living, including burrowing and nesting, at both 3.5 and 7 months compared to control mice (*p* < 0.01). Dual Tg mice built very poor nests, indicating that non-cognitive tasks are also impacted by AD. Overall, dual Tg mice demonstrated behavioral deficits earlier than those shown by the htau mice. In the brain, dual Tg mice had significantly less free Zn compared to control mice in both the dentate gyrus and the CA3 of the hippocampus (*p* < 0.01). Dual Tg mice had increased tangles and plaques in the hippocampus compared to htau mice and the dual Tg mice given Zn water displayed increased tangle pathology in the hippocampus compared to htau mice on Zn water (*p* < 0.05). The dual Tg mouse described here displays pathology reminiscent of the human AD condition and is impaired early on in both cognitive and non-cognitive behaviors. This new mouse model allows researchers to assess how both amyloid and tau in combination impact behavior and brain pathology.

## Introduction

Alzheimer's disease (AD) is a neurodegenerative disorder affecting the lives of an estimated 5.7 million individuals in the United States; this estimate is projected to more than double by the year 2050 (Alzheimer's Association, 2018). The pathological hallmarks of the disease include accumulations of amyloid beta (Aβ) plaques and neurofibrillary tau tangles (NFTs) in the brain. Amyloid and tau accumulation cause deficits in memory, learning, and overall behavior, in a progressive fashion with age. There is currently no cure for AD, only therapeutics that temporarily mitigate symptoms. With this in mind, researchers in the field have formulated numerous hypotheses, speculating which protein species may be playing more prominent roles in AD's progression and the best way to combat it. The amyloid cascade theory proposes that amyloid interacts with tau and exacerbates the deficits (Bloom, 2014). However, tau may also increase the toxic effect of amyloid (Ittner and Götz, 2011). Another theory, the metal hypothesis of AD (Bush, 2013; Adlard and Bush, 2018) is based on the dyshomeostasis of metal species, including Zn, seen in AD. An imbalance of biometals can have major impacts on cognition and on the aggregation of amyloid and tau. The innovative mouse model discussed below can be used to examine these theories.

Transgenic mouse models allow researchers to study the key pathological hallmarks of AD and how they lead to altered cellular responses (e.g., oxidative stress, inflammation) and interact and impact one another (LaFerla and Green, 2012). Although more mouse models exist that focus on mutations in human amyloid precursor protein (hAPP), recently, the importance of tau has received more attention. A meta-analysis assessing studies using the 3xTg-AD mouse [which contains amyloid and tau pathology, as well as mutations in Presenelin-1 (PS1)] showed that tau plays more of a role in cognitive decline than Aβ (Huber et al., 2018). In addition, drug research focusing on Aβ has consistently failed to produce significant clinical findings (Doig et al., 2017; Honig et al., 2018), leading to no new drugs on the market since the early 2000s.

Tau mouse models have helped characterize the dysfunction that NFTs can have on mouse behavior and cellular processes, even though in AD no mutation in tau directly leads to the disease (Kitazawa et al., 2012). However, these mice, when used in conjunction with APP mutations, help to provide a better model for the human AD condition. Examples of mouse models modeling both amyloid and tau pathology include the Tg2576/JNPL3 cross (Lewis et al., 2001), crosses of the Tg2576 and VLW(tau) mice (Pérez et al., 2005; Ribé et al., 2005), hAPP (Swe)/wildtype human Tau (Chabrier et al., 2014), hAPP_NLI_/Tau(P301L) (Paulson et al., 2008), APP23/B6P301L (Bolmont et al., 2007), APP-V717I/TauP301L (Terwel et al., 2008), and the 3xTg-AD mouse (Oddo et al., 2003). These models display both amyloid and tau pathology, with the 3xTg-AD mouse including mutations in PS1.

Despite the fact that no mouse can truly recapitulate every aspect of the human AD condition, mice that bear mutations in both amyloid and tau can help guide researchers in the correct direction and aid in therapeutic research. A summary of known mouse models containing both amyloid and tau pathology is highlighted in Table 1 which shows behavioral impairments and brain pathology previously observed in these mice. These dual pathological mice (those containing both amyloid and tau mutations together) aid in the understanding of how Aβ and tau interact to cause behavioral and cellular damage; however, often more emphasis has been placed on brain pathology rather than behavioral effects.

**Table 1:** Characteristics of selected genetically modified mice used in Alzheimer's disease studies.

**(A)** Background, behavior assessed, and age tested of selected genetically modified mice.			
**Model**	**Background**	**Behavior**	**Age**
**J20** × **rTg4510 - Dual Tg mice**	Mixed C57BL/6J; 129S6*FVB/N	Behavioral disinhibition (increased time in open arms of EZM and decreased time in center of OF) Hyperactive phenotype Increased latencies to find escape hole and spent less time in target quadrant with age - BM	3.5 and 7 months Brains assessed at 8 months
**J20 (hAPP(SweInd))** Alzforum.org/research-models/j20-pdgf-appswind Mucke et al., 2000 Wright et al., 2013	C57BL/6	Wright et al. (2013): impaired spatial reference memory (Radial Arm Maze) Hyperactivity; no differences in anxiety (contextual Fear Conditioning) No BM assessment	Wright et al. (2013): 6, 12, 24, 36 weeks
**rTg4510 (Tau P301L)** Alzforum.org/research-models/rtgtaup301l4510 Ramsden et al., 2005; Santacruz et al., 2005	Mixed 129S6 (activator) × FVB (responder)	Spatial memory deficits by 4 months Hyperexploratory phenotype 2.5–4 months (and beyond) Decreased time in target quadrant Increased errors in the BM Hunsberger et al., 2015	2.5–4 months, various ages (in characterizing) 4–10 months
**3xTg-AD** Alzforum.org/research-models/3xtg Oddo et al., 2003	C57BL/6, 129X1/SvJ, 129S1/Sv	Huber et al., 2018 - meta analysis of studies using only 3xTg-AD mice (51 sources pooling data from MWM and NOR tests) Stover et al., 2015 – Barnes Maze: increased errors and less time in the target zone; no difference in NOR	6.5 months (Stover et al., 2015—NOR and BM)
**JNPL3** × **Tg2576** Alzforum.org/research-models/tg2576 alzforum.org/research-models/jnpl3p301l Lewis et al., 2001 (Tg2576 × JNPL3)	Mixed C57BL/6, DBA/2, SW B6;SJL	None assessed	2.5–3.5, 6–7, 8.5–15 months
**VLW** × **Tg2576** Lim et al., 2001 (VLW mice) Pérez et al., 2005 (VLW × Tg2576) Ribé et al., 2005 (VLW × Tg2576)	Mixed C57BL/6 × SJL; C57BL/6J × CBA	Pérez et al., 2005: none Ribé et al., 2005: MWM - visible platform training VLWxTg2576 mice had longer latencies to escape; at 16 months VLWxTg2576 mice had longer latencies across invisible platform testing days No BM assessment	9, 16, 25 months
**rTg369AB (hAPP**_NLI_ + **Tau (P301L))** Paulson et al., 2008	Activator (CaMKIIa mice) (129S6 (activator) × FVB/N (responder)) Tg3696AB responders (mice with APP and tau mutations) (Tg3696A × Tg3696B)	None assessed	4 months to assess plaque and NFT pathology 13 months used as a time point to study pathology in age-dependent manner
**hAPP(Swe)** × **Tau(Wildtype/non-mutated human)** Chabrier et al., 2014	Multiple founder lines (hAPP + WT human tau) insertion of transgenes into C57BL/6J embryos	Moved significantly more in the open field No preference in the NOR MWM: after 4 days of training, increased latency to find platform compared to nontransgenic mice No BM assessment in hAPP/htau mice	Animals raised for 8 months before behavioral testing 18 months for some biochemical assays (plaques)
**APP23** × **B6/P301L** Bolmont et al., 2007	C57BL/6J B6/P301L (JNPL3 background crossed to B6)	None assessed	Various ages (> 20 months) *F* (21.6 ± 0.9 months) M (27.6 ± 0.7 months)
**APP-V717I** × **Tau P301L** Terwel et al., 2008	FVBN	Open field—no increased anxiety Light/dark box—no data presented Passive inhibitory avoidance—decreased latency than WT Object recognition test—significantly less than WT Conditioned taste aversion—no difference from WT mice	4–6 months (behavior) 14–17 months (histological measures)
**(B)** Brain pathology in selected genetically modified mice.			
**Model**	**Plaques**	**Tangles**	**Other impairments**
**J20** × **rTg4510 - Dual Tg mice**	Plaques detected at 8 months (Congo Red) in the HP	Tangles present and numerous in the HP Tangles exacerbated by chronic zinc administration (Thioflavin-S) Similar AT8 signal to single tau mice	Increased GFAP expression compared to promoter control mice Deficits in non-cognitive activities of daily living measures seen both early and later in disease progression
**J20 (hAPP(SweInd))** alzforum.org/research-models/j20-pdgf-appswind Mucke et al., 2000; Wright et al., 2013	Amyloid deposition (5–7 months) 8.5–15 months—numerous plaques noted	Absent	Increase in gliosis throughout aging; correlates with loss of cells (6-9 months)
**rTg4510 (Tau P301L)** alzforum.org/research-models/rtgtaup301l4510 Ramsden et al., 2005; Santacruz et al., 2005	Absent	Hippocampus by 5.5 months Cortex by 4 months	Progressive neuronal loss and loss of brain weight; forebrain atrophy by 10 months
**3xTg-AD** alzforum.org/research-models/3xtg Oddo et al., 2003	Extracellular Aβ by 6 months	Aβ precedes tau pathology no tau pathology at 6 months Extensive tau in HP CA1 by 12 months	Caruso et al., 2013 increased GFAP by 7 months; at 24 months, still increased GFAP compared to WT
**JNPL3** × **Tg2576** alzforum.org/research-models/tg2576 alzforum.org/research-models/jnpl3p301l Lewis et al., 2001 (Tg2576 × JNPL3)	Plaques detected at 6 months 8.5–15 months, numerous plaques noted	NFTs consistently present and numerous	Motor impairments in TAPP and JNPL3 mice pathology noted in the spinal cord
**VLW** × **Tg2576** Lim et al., 2001 (VLW mice) Pérez et al., 2005 (VLW × Tg2576) Ribé et al., 2005 (VLW × Tg2576)	Scarce amyloid deposits at 9 months Plaques in the HP, amygdala, and cortex in single APP and VLW × Tg2576 mice Sex differences in amyloid load (M < F)	nonsignificant increase in AT8 signaling increase in Ser262 phosphorylation Pérez et al., 2005 16 months - increased Aß in various regions comapred to Tg2576 mice	Decreased neurons at 9 months in the entorhinal cortex 16 months observed cell loss in the CA1 and entorhinal cortex between VLW × Tg2576 mice and wildtypes
**rTg369AB (hAPP**_NLI_ + **Tau (P301L))** Paulson et al., 2008	Plaques in frontal cortex at 4 months APP expression in forebrain By 13 months, great accumulation of plaque pathology	Labeling of neurons positive for changes in phosphorylation and conformation (4 months) 11 months: AT-8 antibody labeled positive neurons (increased at 13 months as well) Mature argyrophilic neurofibrillary tangles by 11 months	Hippocampal and cortical neuronal loss (between 11 and 13 months) Increase in GFAP positive astrocytes between 11 and 13 months
**hAPP(Swe)** × **Tau(Wildtype/non-mutated human)** Chabrier et al., 2014	Contributing hAPP mice had no plaques at 18 months comapred to 3xTg mice (no congo red stained) increases in APP and Aß (soluble and insoluble)	Total tau increased in HP in hAPPxTau mice (no difference in various kinase levels)	Dendritic spine reduction (loss in spine density)
**APP23** × **B6/P301L** Bolmont et al., 2007	Stereological amyloid load no different than APP23 mice	Increased tau pathology (AT8) after amyloid deposition Increased AT8 in entorhinal cortex and hippocampus	Congophilic plaques surrounded by activated glial cells
**APP-V717I** × **Tau P301L** Terwel et al., 2008	Progressive extracellular deposits (10–18 months) IHC detected plaques; amyloid pathology present around 10–12 months prior to tau	Fibrillar tangles detected in the cortex (14–17 months; mAb AT100) Tangles detected in CA1 (13–18 months); different than P301L mouse tau pathology	Vascular amyloid (17 months) Less mobile but not motor impaired Euthanasia of mice between 13 and 18 months for ethical concerns

Cognitive aspects of behavior have received the most attention in various mouse models of AD, due to the well-documented impairments seen in memory. However, non-cognitive behaviors are also impacted in AD (Marshall et al., 2012) and can interfere with the quality of life in both patients and caregivers (Opara, 2012). Measures of daily living in mice can be examined by tests such as burrowing and nesting, which are simple to run and help to provide researchers with non-cognitive measures of wellness that accompany changes in cognition (Deacon, 2012; Jirkof, 2014). Therefore, in assessing a battery of behaviors in AD mouse models, it is important to include these non-cognitive measures.

Alzheimer's pathology in the brain is characterized by the presence of amyloid plaques and tau tangles. Tau is a microtubule-associated protein (MAP) whose major role involves microtubule structure stability and assembly. Tau has numerous possible phosphorylation sites and disruption in tau's phosphorylation state can result in neurofibrillary degeneration, aggregation, and decreases in microtubule stabilization (Spires-Jones et al., 2009; Wang et al., 2013). It is hypothesized that in AD, soluble amyloid hyperphosphorylates tau, leading to disruption in microtubule dynamics, damaging of cellular axonal transport, and a decrease in proteasome activity (Iqbal et al., 2009; Wang et al., 2013). However, the relationship may be more complex, with tau also increasing amyloid's toxicity. In a pathological situation hyperphosphorylated tau is found in the dendrites as well as the axons. Here it can interact with the tyrosine kinase FYN, allowing FYN to phosphorylate NMDA receptors which interact with the post synaptic density 95 (PSD95), leading to excess calcium influx (Ittner and Götz, 2011). But amyloid itself can cause excitotoxicity (Boehm, 2013; Esposito et al., 2013; Reiss et al., 2018) and if tau is unable to bind to FYN, amyloid toxicity is reduced, as shown in tau^−/−^ (knockout) mice (Leroy et al., 2012). Thus, while amyloid may initially cause tau hyperphosphorylation, tau may at a later stage increase amyloid's toxicity. Evidence is conflicting as to whether this also leads to an increase in amyloid. Certain hAPP/tau mice such as the JNPL3 × Tg2576 cross (Lewis et al., 2001) and the APP23/B6P301L (Bolmont et al., 2007) showed no difference in plaque load compared to the sole hAPP mice while other models showed an increase in amyloid pathology (Ribé et al., 2005). Although both Aβ and tau pathology are noted in the AD brain, the progression of tau pathology specifically has been shown to better correlate with dementia than Aβ (Braak and Braak, 1991; Ingelsson et al., 2004; Morris et al., 2014). The interaction of amyloid and tau has been hypothesized to be an essential aspect of AD (Götz et al., 2001; Lewis et al., 2001; Hurtado et al., 2010).

Zinc plays critical roles in normal physiological and neurological functioning. It is abundant in numerous regions of the brain including the hippocampus, amygdala, and the cortex: all of which are affected by AD pathology. In development, Zn is essential for various vital processes (Sandstead, 2003; Gower-Winter and Levenson, 2012). However, an excess of Zn has been shown to be toxic to neurons *in vitro* (Yokoyama et al., 1986) and can lead to an indirect deficiency in Cu (Maret and Sandstead, 2006), which can lead to iron anemia. Levels of Zn should also be monitored in the elderly population. Zinc deficiency has been observed as a result of nutritional intake and inability to obtain appropriate micronutrient levels (Nuttall and Oteiza, 2014). Although supplementation with Zn has been shown to be beneficial in those with advanced age-related macular degeneration (Age-Related Eye Disease Study Research Group, 2001), supplementation without consideration for other metal species can potentially lead to unforeseen negative consequences in elderly patients, such as high levels of Zn in denture creams resulting in decreased levels of Cu (Nations et al., 2008).

Within the AD brain, a dyshomeostasis of metal ions has repeatedly been seen; particularly in Zn, Copper (Cu), and Iron (Fe), all of which have been found in and around plaque pathology (Ayton et al., 2013; Greenough et al., 2013) and disturbances in their levels are noted in AD (Graham et al., 2014). Zinc in particular is a metal of interest for those studying AD, as increases in Zn^2+^ are seen in Aβ plaques and Zn interacts with Aβ peptides (Bush et al., 1994; Barnham and Bush, 2014). As Zn^2+^ is often coreleased with glutamate in neuronal signaling, the extracellular Aβ peptide can bind this free Zn^2+^, promoting its aggregation and assembly into toxic Aβ oligomers (Watt et al., 2010), and the folding into typical β-sheet conformations (Yang et al., 2000). Due to this binding, less Zn^2+^ is brought back into the presynaptic terminal. The use of metal protein attenuating compounds, clioquinol (CQ) (White et al., 2006) and PBT2 have been used to try and reverse this situation; clinical trials utilizing PBT2 and CQ have led to some significant improvements in cognition (Adlard and Bush, 2018).

Zinc also interacts with various kinases and phosphatases impacting tau microtubule stability and assembly, thus affecting tau's phosphorylation state, its conformation, and ultimately its ability to function (Cuajungco et al., 2000; Huang et al., 2014). Studies done *in vitro* have shown that an increase in tau's phosphorylation can be blocked by CQ (Sun et al., 2012; Xiong et al., 2013).

Although *in vitro* studies are valuable to assessing the impact of Zn on tau and Aβ, changes in the levels of one metal can affect the levels of others *in vivo* (Maret and Sandstead, 2006; Nations et al., 2008) The use of animal models allows for more translational findings and the ability to see the impact that Zn has in conjunction with AD pathology.

Administration of Zn to mouse models of AD has been shown to have both adverse and positive effects. Zinc administration at 10 ppm through the drinking water has been shown to impair spatial memory in the CRND8 mouse (Flinn et al., 2014), Tg2576 mouse (Linkous et al., 2009; Railey et al., 2011), and rTg4510 mouse (Craven et al., 2018). APP/PS1 transgenic mice also display deficits in spatial memory after Zn administration through the drinking water (Wang et al., 2010). In contrast to these results, Zn administration has been seen to prevent spatial memory deficits in the Morris Water Maze (MWM) and increase BDNF brain levels in the 3xTg-AD mouse (Corona et al., 2010) and decrease amyloid burden in the Tg2576 mouse (Harris et al., 2014); however, these studies have used higher levels of Zn compared to previous research and have used different sources of Zn (i.e., acetate and sulfate).

In the present study, a mouse with both amyloid and tau pathology is introduced, herein referred to as the dual Tg mouse. This mouse was bred from two common mouse strains that have yet to be crossed in the AD literature, the rTg4510 tau mouse and the J20 hAPP mouse. The offspring were characterized through a battery of behavioral tests, including both cognitive and non-cognitive measures. These tests were selected to assess general locomotion, anxiety and risk-taking behavior, spatial memory, and depressive behavior. In addition, activities of daily living (ADL), such as burrowing and nesting, were measured. Half of the mice were supplemented with Zn through the drinking water to assess the impact chronic Zn administration would have on overall behavior and on biochemical analyses. Brains were analyzed for glial fibrillary acidic protein (GFAP) (a neural marker of inflammation), free Zn, and plaque and tangle pathology.

We hypothesized that this mouse, containing both mutated amyloid and tau, would display deficits in spatial memory and ADL, have increased anxiety and depressive-like behaviors, and display higher levels of GFAP, as well as an increase in the number of tangles and plaques compared to (a) WT mice with no mutations, (b) tTA under CaMKIIa promoter mice (herein referred to as tTA mice), and (c) htau mice. Zinc was hypothesized to worsen both the behavioral deficits and brain parameters. tTA mice were included as a comparison group to rule out whether the expression/promoter system had any effect on behavior and pathology.

The results showed that dual Tg mice displayed impairments in spatial memory, demonstrated behavioral disinhibition, had increased locomotion in the forced swim test, and had deficits in daily living measures compared to both control and htau mice. Although the htau mice also showed some deficits in these parameters, the dual Tg mice showed greater brain pathology than htau mice and this pathology was affected by zinc. This mouse serves as an appropriate model to explore the impact of both amyloid and tau in AD.

## Methods

### Animals

Mice were bred through pairing the J20 hAPP mouse (Tg(PDGFB-APPSwIND)20Lms/2Mmjax) and the rTg4510 tau mouse (Tg(Camk2a-tTA)1Mmay Tg(tet0-MAPT^*^P301L)#Kha/J) ordered through the Jackson Laboratory (Bar Harbor, ME). Male J20 hAPP mice and female rTg4510 mice were used for breeding purposes. Animal genotype was confirmed through analysis of tail snips by Transnetyx, Inc. (Cordova, TN) Animals were then housed in separate cages (Animal Care Systems) based on sex and genotype. Mice containing both mutated APP and tau (along with the tetracycline transactivator (tTA) under the control of a CaMKIIa promoter) are referred to as dual Tg mice while those containing mutated tau (along with the tetracycline transactivator (tTA) under the control of a CaMKIIa promoter) are referred to as htau mice. Animals with only tTA, or no mutations present (wildtype - WT) were kept and run as controls. These were the genotypes of interest in the given study. Controls in the context of this study generally refer to both tTA and WT mice, but the individual group is included in the results section if *p-*values differed. Mice bred for this study were of an F1 generation.

### Housing

Each amber, polysulfone cage (Animal Care Systems) was equipped with both an igloo and an igloo with an attached running wheel. Nylon bones were supplied for the mice to chew on. The colony was maintained on a 12-h light/dark cycle. Mice were handled weekly to acclimate to human touch for behavioral testing. Animals were provided food and water *ad libitum*. All procedures were approved by the George Mason University Institutional Animal Care and Use Committee.

### Zinc water

At 8 weeks of age, all mice began to receive water through plastic water bottles. Half of the animals received normal laboratory tap water whereas the other half received water formulated as 10 parts per million (ppm) Zn *ad libitum*. Zinc water was prepared using a 10,000 ppm solution of Zn dissolved in 5% nitric acid (Assurance SPEX CertiPrep) and brought to a pH of ~7 using sodium carbonate; this is in accordance with prior studies from our lab (Linkous et al., 2009; Railey et al., 2010; Craven et al., 2018). Metal levels were verified by the United States Geological Survey (USGS, Reston, VA). Water was replaced every 2 weeks, shaken routinely, and was continually monitored by Krasnow Animal Facility staff.

### Behavioral tests and measurements

Mice were taken from their home cages and placed into individual cages on testing days. Animals were tested at two time points: 3.5 and 7 months of age. These testing points were chosen to assess pathology at both early and later stages of disease progression. Behavioral tests animals underwent included the open field test (OF), elevated-zero maze (EZM), Barnes maze (BM), forced swim test (FS), and measures of daily living (burrowing and nesting). A camera positioned above the testing apparatus connected to a central computer running TopScan software (CleverSys, Inc.) was used for OF, EZM, and BM. Research assistants blind to animal conditions scored activity in the FS test. Between each animal tested, the respective apparatus was cleaned with 70% ethanol to minimize olfactory cues. For measures of daily living, mice were individually housed in shoe-box cages for 2 days with access to food and water *ad libitum* (as discussed below). The number and sex of the animals used for behavioral analysis and the forced swim test specifically are given in Table 2A. Table 2B shows the order of behavioral tests run at both 3.5 and 7 months of age.

Table 2Number of animals used in current experiment.

**Table:** 

**(A)** Number of animals used in behavioral testing.				
	**Dual Tg**	**htau**	**tTA**	**Wildtype (no mutations)**
Lab water	10 **(7)** 5M, 5F	11 **(10)** 4M, 7F	12 7M, 5F	10 5M, 5F
Zinc water	11 **(10)** 5M, 6F	12 5M, 7F	12 7M, 5F	11 6M, 5F

*Over the course of the experiment, 4 dual Tg mice and 1 htau mouse died before completion of the forced swim test at 7 months. The N for each group for FST analysis is denoted in **(bold)**. Eight dual Tg lab animals were analyzed for burrowing and nesting assays*.

**Table:** 

**(B)** Animal testing order.									
**3.5 months**	**One day**	**One day**	**Eight days**	**One day**	**One day**	**Nine days**	**One day**		
	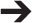	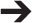	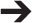	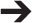	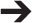	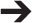	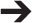		
	OF	EZM	BM	Burrowing	Nesting	Circadian rhythm	FS		
									Aged until second testing period
**7 months**	**One day**	**One day**	**Eight days**	**One day**	**One day**	**Nine days**	**One day**	**Animals**	
	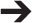	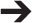	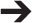	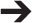	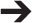	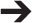	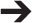	**euthanized**	
	OF	EZM	BM	Burrowing	Nesting	Circadian rhythm	FS

**Table:** 

**(C)** Sample size per group for tissue analysis.				
	**Dual Tg**	**htau**	**tTA**	**Wildtype (no mutations)**
**LAB WATER**				
WB:	4	4	4	3
ZP-1:	4	2	3	3
TS:	5	4	3	3
CR:	6	2	2	2
**ZINC WATER**				
WB:	4	4	4	3
ZP-1:	3	3	3	3
TS:	3	4	3	3
CR:	5	2	2	2

*Animal numbers for genotype group and water conditions for various biochemical tests. WB, Western Blot; ZP-1, Zinpyr-1 fluorescence; TS, Thioflavin-S; CR, Congo Red. ZP-1fluorescence was done on whole brains and when possible, right hemispheres from animals that had their left hemisphere homogenized for WB analysis were used for other histological tests (TS, CR)*.

### Open field (OF)

The open field (OF) test is used to assess locomotion and anxiety. The OF apparatus consisted of two square Plexiglas boxes that each measured 41 × 41 cm. Two animals were run at a time and each animal was given one 5-min trial; each trial began with the mouse placed facing the back wall in the surround. Percent time spent in open/surround sections, total distance traveled, and latency to enter the center region were measured.

### Elevated zero maze (EZM)

The elevated zero maze (EZM) is used to assess anxiety and risk-taking behavior. The EZM apparatus is an “O” shaped platform that is raised off the ground and divided into two sections with 31 cm-high walls (closed sections) and two sections with no surrounding walls (open sections). Each animal was given a single 5-min trial that began with the animal being placed in a closed section, head facing away from the open section. An animal was considered in an open or a closed section when all four of the paws were in that particular section. Percent time spent in open/closed arms and number of head dips were measured.

### Barnes maze (BM)

The Barnes maze (BM) is a test of spatial memory. The BM is a circular platform with various holes along the perimeter including one hole that leads to an escape box containing bedding. Spatial cues that the animals use to learn the location of the escape box were positioned around the maze. The BM apparatus had 40 holes and the overall method used was similar to that used previously (Flinn et al., 2014). On the first day of testing, each mouse was placed in the center of the maze under a dark cup and then guided to the escape box; once in, the mouse was allowed to stay for 30 s and was placed back into its testing cage for 2 min before undergoing a second trial (2 min inter-trial interval, ITI). This first day served as habituation to the testing apparatus; days 2–6 served as acquisition days. Each animal was given three trials with an ITI of 15 min. If the escape hole was not found within 3 min, the mouse was gently guided to the hole and allowed 30 s in the hole. Bedding was replaced after each trial. Latency to find the escape hole, time spent in the target quadrant, and number of primary errors were analyzed. Primary errors were defined as the number of holes explored prior to finding the escape hole for the first time. On the 7th day of the BM paradigm, the escape box was removed, and each animal was given one trial and the time spent in the target quadrant was recorded. Between testing periods (3.5 vs. 7 months), the escape box was moved to a different quadrant and cues were randomized to new locations to prevent any possible carry-over effects.

### Activities of daily living (ADL)

#### Burrowing

Daily living measures included analysis of burrowing and nesting. Mice were individually housed to assess ADL measures; burrowing behavior was analyzed first. Burrowing is a non-cognitive action seen in rodent species; this innate digging aids in finding/forming a shelter and storing food away from predators (Deacon, 2006). Mice were individually housed in cages with PVC pipes containing 250 grams of pea-gravel (small rocks) with one end closed off. Measurements of the amount of pea-gravel buried were taken at two time points: after 2 h and overnight. The 2-h measurement is thought to be more sensitive of a measure (Deacon, 2006) and is the measurement reported in this paper. After weighing the PVC tube at the 2-h mark, the tube was carefully placed in the same location, to avoid disturbing the pea-gravel already buried.

#### Nesting

After the burrowing assay, the bedding was replaced with corncob bedding (as opposed to standard bedding which mice could use to make nests) and three grams of shredded paper were dispersed throughout the cage. Pictures were taken after 24 h and scored by raters blind to the animals' group on a scale of 1–3. A score of 1 represented no nest constructed and 3 represented a fully formed nest, with all of the paper in the cage being gathered in one area. Intra-class correlations were calculated on all the nesting scores gathered from the same two raters to ensure reliability.

### Forced swim (FS)

The forced swim (FS) test is considered a test of depression. Mice were placed into a container filled with 23–25°C water and the time to become immobile (latency to immobility) and time spent immobile were measured. Each FS trial lasted a period of 6 min: the first 2 min served as habituation. Although the first 2 min served as habituation, this time was considered in measuring latency to immobility. The last 4 min were analyzed for time spent immobile. Two animals were run at once (in two containers) with a barrier between the tubes with research assistants blind to condition timing latencies to immobility and bouts of inactivity. After each trial, animals were dried off and placed briefly under a heat lamp to prevent hypothermia. Each tube of water was emptied, and fresh water was added and checked for correct temperature before the next trial. Mice that swam for the entire testing period were assigned the maximum value for latency [360 s (total length of the FS trial)] and the minimum for time spent immobile (0 s)].

### Brain analysis

Twenty-four hours after the completion of the FS test at the 7-month testing period, animals were euthanized by isofluorane overdose and sacrificed by guillotine, confirming death. Brains were then extracted. A random sample of mice were selected for western blot analysis. These mice had their left hemisphere homogenized on the day of extraction and the right hemisphere was placed in dry ice for future histological analysis. Animals that were not selected for western blot analysis had their whole brain placed into dry ice and then placed into a −80°C freezer for future processing. Fresh-frozen brain analysis was preferred over perfusion since certain measures (Zinpyr-1 analysis) will not yield reliable data using perfused tissue. Western blot analysis was done on left hemisphere lysates, and Zinpyr-1 (ZP-1) free zinc fluorescence, Thioflavin-S staining, and Congo Red amyloid plaque staining were carried out primarily on whole brain sections. The numbers for each biochemical analysis run are provided in Table 2C.

#### Western blotting

The left hemispheres of randomly selected animals (Table 2C) were homogenized in ice-cold RIPA buffer with Halt™ protease/phosphatase inhibitor cocktail (ThermoFisher) according to manufacturer's instructions at time of extraction using a glass dounce tissue grinder (Sigma Aldrich). Samples were then centrifuged using a TOMY MTX-150 High speed micro refrigerated centrifuge at 14,000 RPM at 4°C for 20 min. Soluble protein concentration was determined through use of the BCA protein assay kit (Pierce™, ThermoFisher). Thirty micrograms of protein for each animal was prepared under reduced conditions and heated at 90°C for 5 min. Samples were then loaded onto NuPAGE™ 4–12% Bis-Tris gels (Invitrogen) and transferred to nitrocellulose membranes using the iBlot 2 gel transfer device (ThermoFisher). Membranes were blocked with 5% non-fat dried milk in PBST (1X PBS with 0.1% Tween 20 (Sigma-Adrich), referred to as: Blotto), for 30 min and incubated with primary antibody overnight at 4°C. Primary antibodies included: GAPDH (1: 2,500, Abcam, ab9485), GFAP (1:1,000, Cell Signaling, D1F4Q), Tau (1: 5,000, Abcam, ab32057), and AT8 (Phospho-Tau Ser202/Thr205) (1: 1,000, ThermoFisher, MN1020). Membranes were then washed in three changes of PBST, blocked for 30 min in Blotto and incubated with an appropriate HRP-conjugated secondary antibody (goat anti-rabbit: 1: 20,000, Abcam, ab6721 or rabbit anti-mouse: 1: 20,000, Abcam, ab97046) for 1 h. Blots were developed using SuperSignal West Pico PLUS Chemiluminescent Substrate (ThermoFisher) and imaged with the G:Box Chemi-XT4 (Syngene) system with GENESys software (V1.2.5.0). Bands were imaged and analyzed through ImageJ (NIH) software.

#### Zinpyr-1 (ZP-1) free zinc fluorescence

Zinpyr-1 was chosen to stain for free Zn^2+^ as it has bright fluorescence and a high affinity for the metal species (Woodroofe et al., 2004). A 1 mM stock solution of Zinpyr-1 (ZP-1, mw 823.72 g) (Abcam, ab145349) was prepared in DMSO; a 17 μM working solution was then made using 0.9% saline. The ZP-1 working solution was made fresh each day of use. Whole brains from selected animals (Table 2C) were sliced coronally at 16 μm on a Leica CM3050S cryostat across the hippocampal region and placed on positively charged slides. Slices were then covered in working ZP-1 solution in darkness for 2.5 min and after were tilted to remove the ZP-1 solution. Sections were immediately imaged using an Olympus BX51 fluorescence microscope equipped with a fluorescein isothiocyanate (FITC) cube, and mercury burner. Images were analyzed at 2x objective with consistent exposure time between samples. All collected images were analyzed with ImageJ (NIH). Images were analyzed by taking average green fluorescence values from the brain regions of interest and subtracting the average background from it. Average green values were collected for the dentate gyrus (DG), CA3, and CA1 regions of the HP. Higher green fluorescence values are indicative of greater amounts of free Zn.

#### Thioflavin-S staining for tangles

Thioflavin-S staining protocol for tangle pathology was adapted from Sun et al. (2002) and has been used previously in our lab (Craven et al., 2018). Brains from selected animals (Table 2C) were sliced at 16 μm and placed into PFA for 8 min and then washed in 1X PBS. Slides were then placed into 0.25% potassium permanganate for 4 min followed by 1% sodium borohydride for 4 min. Slides were rinsed in distilled water and placed in 0.05% Thioflavin-S (Sigma Aldrich) prepared in 50% ethanol. Slides then underwent two different exchanges of 80% ethanol and then were dipped in three exchanges of distilled water. Slides were placed in 10X PBS in the refrigerator for 30 min, rinsed in distilled water, and then imaged. Images were taken using an Olympus BX51 fluorescence microscope with a FITC cube under a 20x objective. Each captured image was analyzed using ImageJ (NIH) software. The image's color threshold was adjusted using the default setting, and analysis of captured particles was adjusted to 100 pixels across all images. Tangles detected on the edge of the image were excluded. Dual Tg and htau mice were the only mice of interest given that they are the sole animals containing mutations that should result in tangle pathology.

#### Congo red amyloid plaque staining

The HT-60 Congo red amyloid stain kit (Sigma-Aldrich) was used to assess presence of plaques in the HP according to manufacturer's instructions. Brains of select animals (Table 2C) were run through the staining procedure. Slides were immediately analyzed for overall plaque count using an Olympus BX51 microscope. Images were analyzed at 20x objective. Dual Tg mice were the only mice of interest given that they are the sole animals containing a mutation that should result in amyloid plaque pathology. Animals of other genotypes were assessed as negative controls.

### Statistical analysis

All values are presented as mean ± SEM. 4 (genotype) × 2 (water) × 2 (sex) × 2 (age) mixed ANOVAs were run for dependent variables assessed in the OF, EZM, FS, and measures of daily living (burrowing and nesting). Simple effects analyses were run to detect differences in genetic groups across ages and as follow-ups to interactions. Pairwise comparisons were assessed following significant main effects. Both tTA and WT are described as controls except when different levels of statistical significance were observed.

Barnes maze analysis: A 4 (genotype) × 2 (water) × 2 (sex) × 5 (acquisition day) mixed ANOVA was run for the following dependent variables at both 3.5 and 7 months: latency to find the escape hole, percent time spent in target quadrant, and primary errors. A 4 (genotype) × 2 (water) × 2 (sex) × 2 (age) mixed ANOVA was run on percent time spent in the target quadrant on the 24-h probe trial (day 7). Significant main effects were followed by *post-hoc* analyses and significant interactions were followed by simple effects analysis.

A 4 (genotype) × 2 (water) × 2 (sex) × 3 (hippocampal region) mixed ANOVA was run for ZP-1 fluorescence values. Two-way ANOVAs (genotype and water as independent variables) were run for dependent variables measured in western blotting and Thioflavin-S staining. All data were analyzed through SPSS v. 19 and graphs were created through GraphPad Prism 7. *P* < 0.05 was considered significant and any main effects were followed up by Bonferroni *post-hoc* analysis. Greenhouse-Geisser corrections were made to correct when sphericity could not be assumed in mixed ANOVA analyses.

## Results

### Genotype distribution

The expected frequency of each genotype in this cross was 1/8. There were no significant differences between expected and obtained frequencies in the breeding of this new mouse: χ^2^(7, *N* = 233) = 0.511, *p* = 0.999. This study examined the dual Tg, htau, tTA, and WT mice. Thus, approximately 50% of the litter yields were used in this study.

### Weights over time

A 4 (genotype) × 2 (water) × 2 (sex) × 7 (month) mixed ANOVA on monthly weight measurements, with the Greenhouse-Geisser correction, revealed a significant effect of month, *F*_(4.992, 374.386)_ = 466.932, *p* < 0.001. In addition, significant genotype × month, *F*_(14.975, 374.386)_ = 8.699, *p* < 0.001, and sex × month, *F*_(4.992, 374.386)_ = 4.876, *p* < 0.001 interactions were noted. Dual Tg mice weighed significantly less than control mice at each month (*p* < 0.05), and htau mice weighed less than both WT (*p* < 0.05) and tTA mice (*p* < 0.01) from months 2 and 4 onward, respectively. Animals on lab water weighed significantly more than those on Zn water at month 2, when they were first put on Zn water, and from month 5 onward (*p* < 0.05). Male mice weighed significantly more than female mice at each time point (*p* < 0.001) (Supplementary Figures 1A–C).

### Brain size

Dual Tg and htau mice had smaller brains than control mice (Supplementary Figure 2).

### Effect of zinc

There were no main effects of zinc or sex on recorded behaviors and thus these are not included in the following analyses. However, there were some significant interactions with zinc and/or sex which were further explored and reported.

### Open field

#### Time spent in the center

**Figure 1:**
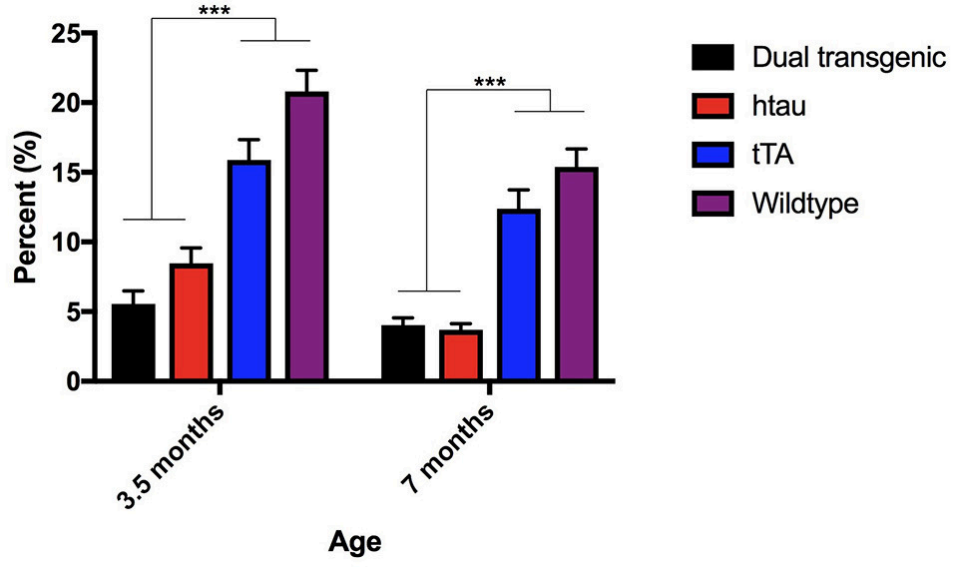
Open field percent time spent in the center. Dual Tg and htau mice spent significantly less time in the center of the OF than control mice at both time points (*p* < 0.001). With increased age, tau (*p* < 0.01), tTA (*p* = 0.01), and WT mice (*p* = 0.001) spent less time in the center of the OF. Bars represent mean ± SEM (***p < 0.001).

There was a significant main effect of genotype, *F*_(3, 73)_ = 65.335, *p* < 0.001 and a significant within subject effect of age, *F*_(1, 73)_ = 23.740, *p* < 0.001. Dual Tg and htau animals spent significantly less time in the center than control mice at both ages tested (*p* < 0.001). With age, htau (*p* < 0.01), tTA (*p* = 0.01), and WT mice (*p* = 0.001) spent less time in the center of the OF (Figure 1).

#### Latency to enter the center

There was a significant effect of genotype, *F*_(3, 73)_ = 12.357, *p* < 0.001 for latency to enter into the center of the OF. Dual Tg and htau animals took significantly longer than control (*p* < 0.001) animals to enter the center at 3.5 months. At 7 months, dual Tg mice took significantly longer to enter the center than tTA (*p* < 0.01) and htau mice took significantly longer to enter than tTA (*p* < 0.001) and WT mice (*p* < 0.01) (Supplementary Figure 3A).

#### Distance traveled in the OF

There was a significant effect of genotype for distance traveled, *F*_(3, 73)_ = 17.931, *p* < 0.001. Dual Tg mice traveled a greater distance than control mice (*p* < 0.001) and htau mice (*p* < 0.01) at 3.5 months and a greater distance than tTA (*p* < 0.01) and WT mice (*p* < 0.001) at 7 months. htau mice traveled a greater distance than tTA (*p* < 0.01) and WT mice (*p* < 0.001) at 7 months and with age, traveled a significantly greater distance (*p* < 0.05) (Supplementary Figure 3B).

### Elevated zero maze

#### Time spent in the open arms

**Figure 2:**
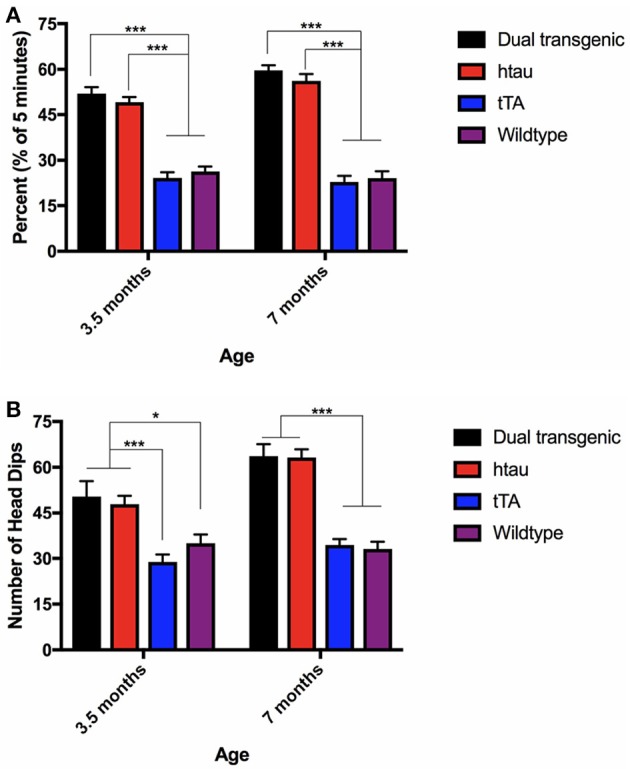
**(A)** Elevated zero maze percent time in the open arms. Dual Tg and htau mice spent significantly more time in the open arms than control animals (*p* < 0.001). With increased age, dual Tg and htau mice spent significantly more time in the open arms of the EZM (*p* < 0.001). Bars represent mean ± SEM (***p < 0.001). **(B)** EZM head dips. Dual Tg and htau mice made significantly more head dips in the EZM at 7 months than at 3.5 months (*p* < 0.001). At both ages, dual Tg and htau mice made significantly more head dips than control mice did. Bars represent mean ± SEM (*p < 0.05, ***p < 0.001).

There was a significant main effect of genotype, *F*_(3, 73)_ = 100.028, *p* < 0.001, a significant within-subject effect of age, *F*_(1, 73)_ = 8.996, *p* = 0.004, and a significant age × genotype interaction, *F*_(3, 73)_ = 6.791, *p* < 0.001. Dual Tg and htau mice spent significantly more time in the open arms of the EZM at 7 months than they did at 3.5 months (*p* < 0.001). Dual Tg and htau animals spent significantly more time in the open arms than control animals at both ages (*p* < 0.001) (Figure 2A).

#### Head dips

Analysis of head dips in the EZM revealed a significant effect of genotype, *F*_(3, 73)_ = 30.137, *p* < 0.001, a significant effect of age, *F*_(1, 73)_ = 22.335, *p* < 0.001, and a significant age × genotype interaction, *F*_(3, 73)_ = 5.103, *p* = 0.003. Dual Tg and htau mice made significantly more head dips in the EZM at 7 months than at 3.5 months (*p* < 0.001). At 3.5 months, dual Tg and htau mice made significantly more head dips than tTA (*p* < 0.001) and WT mice (*p* < 0.05). At 7 months, dual Tg and htau mice made significantly more head dips than control mice (*p* < 0.001) (Figure 2B).

### Barnes maze

#### Latency to find the escape hole

There was a significant effect of day at both 3.5, *F*_(4, 292)_ = 3.250, *p* < 0.05, and 7 months *F*_(4, 292)_ = 3.282, *p* = 0.012. At 3.5 months, there was a significant effect of genotype, *F*_(3, 73)_ = 15.155, *p* < 0.001, and a significant sex × water interaction, *F*_(1, 73)_ = 4.739, *p* < 0.05. Wildtype mice were significantly faster at finding the escape hole than dual Tg and htau mice (*p* < 0.001) and tTA mice (*p* = 0.006). Male mice on Zn water took longer to find the escape hole than did males on lab water (*p* = 0.013) (Supplementary Figure 4).

**Figure 3:**
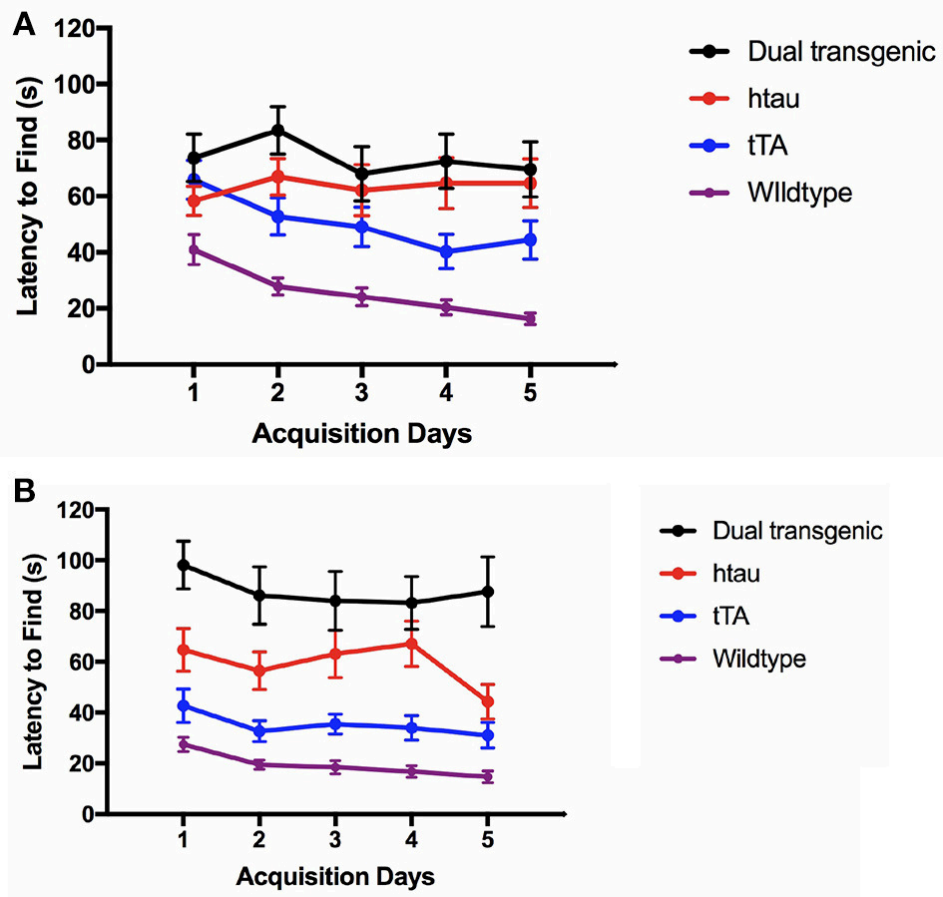
**(A)** Latency to find the escape hole (3.5 months). At 3.5 months, WT mice found the escape hole significantly faster than dual Tg and htau mice (*p* < 0.001) and tTA mice (*p* < 0.01). Bars represent mean ± SEM. **(B)** Latency to find the escape hole (7 months). At 7 months, dual Tg mice took longer to find the escape hole than control (*p* < 0.001) and tau mice (*p* = 0.004). Tau mice took longer to find the escape hole than WT mice (*p* < 0.001), and tTA mice (*p* < 0.05). Bars represent mean ± SEM.

At 7 months, there was a significant effect of genotype, *F*_(3, 73)_ = 27.590, *p* < 0.001. Dual Tg mice took longer to find the escape hole than control (*p* < 0.001) and htau mice (*p* = 0.004). htau mice took longer to find the escape hole than WT mice (*p* < 0.001) and tTA mice (*p* < 0.05) (Figures 3A,B).

#### Percent time spent in the target quadrant

There was a significant effect of day at both 3.5 months, *F*_(4, 292)_ = 7.599, *p* < 0.001, and 7 months, *F*_(4, 292)_ = 11.238, *p* < 0.001. At both ages, there was a significant effect of genotype, *F*_(3, 73)_ = 16.222, *p* < 0.001 (3.5 months); *F*_(3, 73)_ = 51.498, *p* < 0.001 (7 months) (Supplementary Figures 5A,B). At 3.5 months, WT animals spent the most time in the target quadrant each day compared to all other groups. Dual Tg mice spent less time in the target quadrant than WT mice (*p* < 0.001). htau mice spent significantly less time in the target quadrant than tTA mice (*p* = 0.003), and WT mice (*p* < 0.001), and tTA mice spent significantly less time in the target quadrant than WT mice (*p* < 0.05). At 7 months, dual Tg and htau mice spent less time in the target quadrant than control mice did (*p* < 0.001). In addition, at 7 months of age, there was a significant sex × water interaction, *F*_(1, 73)_ = 6.926, *p* = 0.01. Male mice on lab water spent longer in the target quadrant than those on Zn water (*p* = 0.029) (Supplementary Figure 5C).

#### Primary errors

There was a significant effect of day at both 3.5 months, *F*_(3.593, 262.289)_ = 7.484, *p* < 0.001 (Greenhouse-Geisser correction), and 7 months *F*_(3.516, 256.688)_ = 13.211, *p* < 0.001 (Greenhouse-Geisser correction) (Supplementary Figures 6A,B). At both time points, there was a significant main effect of genotype, *F*_(3, 73)_ = 3.553, *p* = 0.018 (3.5 months); *F*_(3, 73)_ = 3.113, *p* < 0.05 (7 months). At 3.5 months, tTA mice made significantly more errors than WT mice (*p* < 0.01). At 7 months, there was a trending difference between WT mice and dual Tg (*p* < 0.10) as well as between WT and htau mice (*p* < 0.10). At 3.5 months, simple effects analysis of a sex × genotype interaction [*F*_(3, 73)_ = 3.134, *p* < 0.05], revealed that female htau mice made significantly more errors than male htau mice (*p* < 0.05). Simple effects analysis of a sex × water interaction [*F*_(1, 73)_ = 4.112, *p* < 0.05], revealed that male mice given Zn water made more primary errors than males on lab water (*p* = 0.05) (Supplementary Figure 6C).

#### Percent time in target quadrant on day 7 (24 h probe)

**Figure 4:**
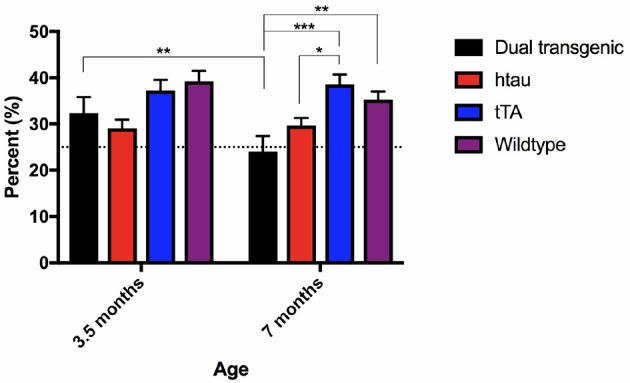
Barnes maze 24-h probe trial. Dual Tg mice spent less time in the target quadrant on the 7th day probe trial than control mice (*p* < 0.01) and htau mice spent less time in the target quadrant than tTA (*p* < 0.01) and WT mice (*p* < 0.05). Dual Tg mice spent significantly less time in the target quadrant with age (*p* < 0.01). Bars represent mean ± SEM (*p < 0.05, **p < 0.01, ***p < 0.001) The dotted line represents chance performance (25%).

There was a significant main effect of genotype, *F*_(3, 73)_ = 7.554, *p* < 0.001 (Figure 4). Dual Tg mice spent less time in the target quadrant than control mice (*p* < 0.01) and htau mice spent less time than tTA (*p* < 0.01) and WT mice (*p* < 0.05). At 3.5 months, htau mice spent less time than WT mice (*p* < 0.05). At 7 months, dual Tg mice spent less time than tTA (*p* < 0.001) and WT mice (*p* < 0.01). hTau mice spent less time than tTA mice (*p* < 0.05). Dual Tg mice spent significantly less time in the target quadrant with age (*p* < 0.01).

In summary, WT mice were fastest at entering and finding the escape hole in the BM task. While there was no difference between dual Tg and htau mice at 3.5 months, at 7 months, dual Tg mice took longer to find the escape hole than did htau mice. Dual Tg mice spent less time in the target quadrant compared to WT mice and worsened with age during the seventh day probe trial. Dual Tg mice also made more primary errors than did WT mice. No main effects of water were seen; however, male mice on Zn water performed significantly worse than those on lab water on several measures.

### Forced swim test

#### Latency to become immobile

**Figure 5:**
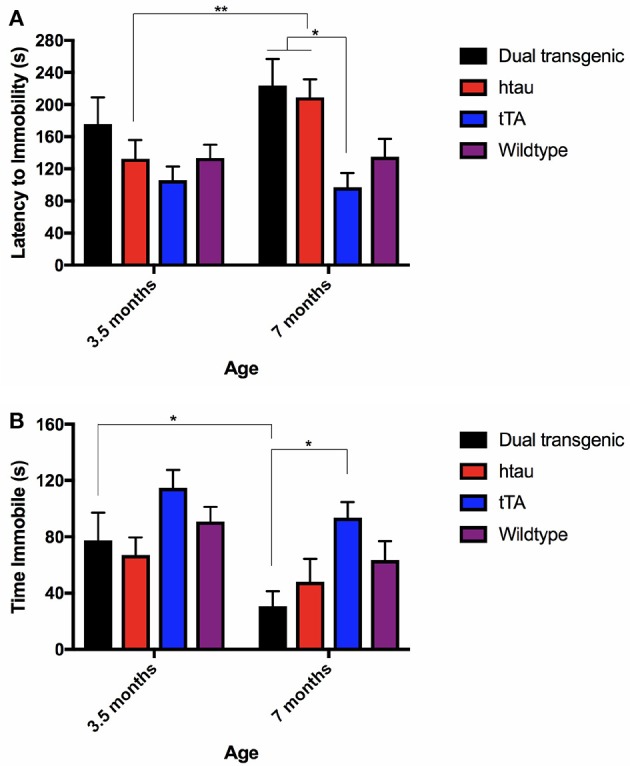
**(A)** FST latency to immobility. htau mice spent more time swimming at 7 months compared to 3.5 months (*p* < 0.01). At 7 months specifically, tTA mice had shorter latencies to become immobile than dual Tg and htau mice (*p* < 0.05). Bars represent mean ± SEM (*p < 0.05, **p < 0.01). **(B)** FST time spent immobile. All genetic groups spent less time immobile; dual Tg mice spent significantly less time immobile (increased activity) with increased age (*p* < 0.05). At 7 months, tTA mice spent more time immobile than dual Tg mice (*p* < 0.05). Bars represent mean ± SEM (*p < 0.05).

There was a significant effect of genotype, *F*_(3, 68)_ = 3.720, *p* < 0.05, and with the Greenhouse-Geisser correction, a significant effect of age, *F*_(1.000, 68.000)_ = 4.345, *p* < 0.05. htau animals spent more time swimming at 7 months of testing than at 3.5 months (*p* < 0.01) (Figure 5A). At 7 months, dual Tg and htau mice took longer to become immobile than tTA mice (*p* < 0.05). Dual Tg mice spent the longest time swimming at both ages.

#### Time spent immobile

There was a significant effect of genotype, *F*_(3, 68)_ = 4.039, *p* < 0.05, and with the Greenhouse-Geisser correction, a significant effect of age was noted, *F*_(1.000, 68.000)_ = 10.91, *p* < 0.01. Dual Tg mice spent significantly more time active with increased age (*p* < 0.05) (Figure 5B). In addition, at 7 months, dual Tg mice spent significantly more time swimming compared to tTA mice (*p* < 0.05).

### Measures of daily living

#### Burrowing behavior

**Figure 6:**
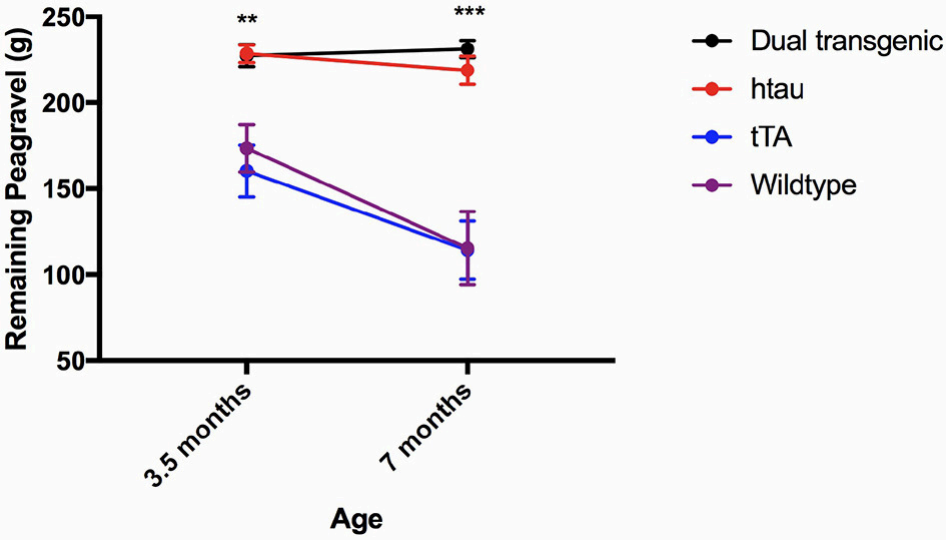
Burrow assessment: 2 h. Dual Tg and htau mice burrowed significantly less pea-gravel at the 2-h measurement mark compared to control mice at both 3.5 (*p* < 0.01) and 7 (*p* < 0.001) months. Control mice burrowed significantly more at the 2 h period at 7 months compared to 3.5 months. Transgenic mice showed a virtual inability to burrow early on (Higher values on the figure refer to amount left in the tube, thus less burrowed/removed).

Analysis of pea-gravel after 2 h showed a significant effect of age, *F*_(1, 71)_ = 16.227, *p* < 0.001, a significant effect of genotype, *F*_(3, 71)_ = 23.493, *p* < 0.001, and an age × genotype interaction, *F*_(3, 71)_ = 4.307, *p* < 0.01. Control mice burrowed significantly more pea-gravel at 7 months of age compared to 3.5 months (*p* < 0.001). Transgenic mice burrowed significantly less pea-gravel than control mice at both 3.5 (*p* < 0.01) and 7 months (*p* < 0.001) (Figure 6).

#### Nesting

**Figure 7:**
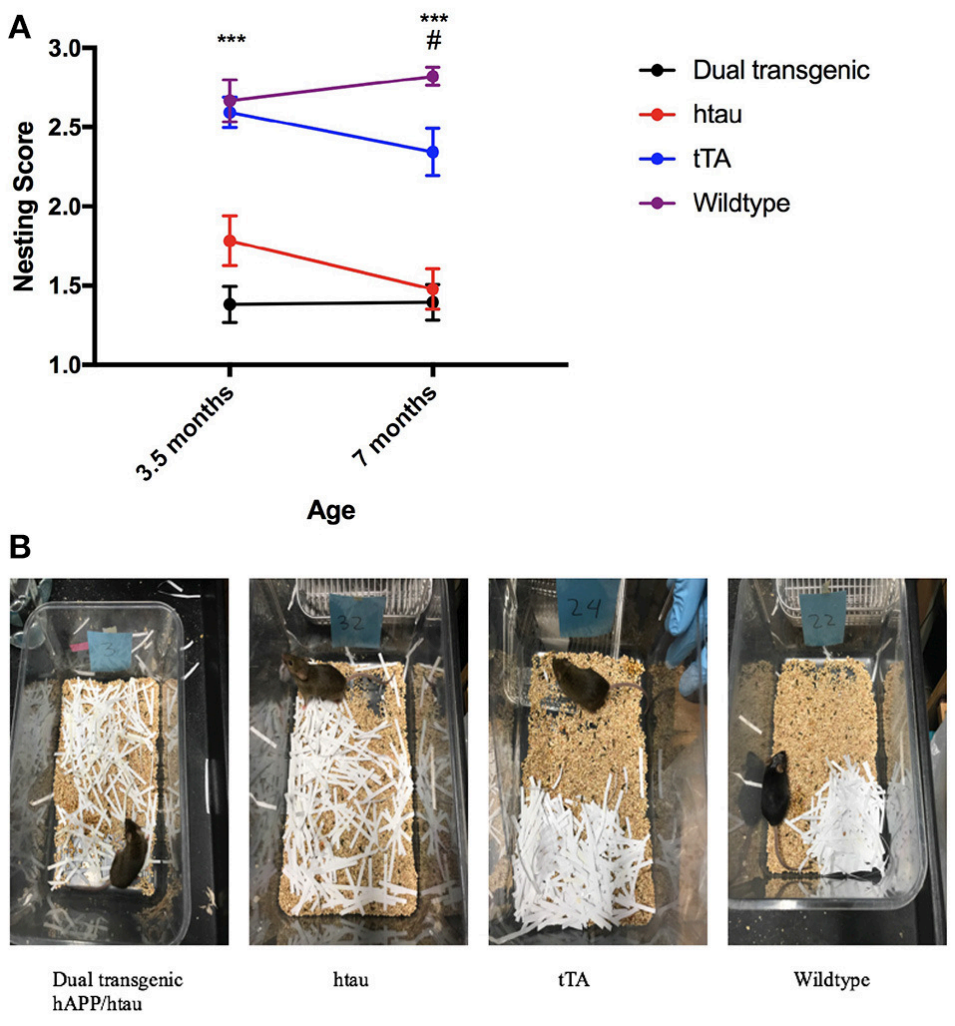
**(A)** Nesting. Dual Tg and htau mice built significantly poorer nests than control mice (***p < 0.001) at both 3.5 and 7 months. With age, tTA mice built significantly worse nests (*p* < 0.05) and at 7 months built significantly worse nests than WT mice (^#^*p* < 0.05). Nesting score: 1 (poorest nest; no nest constructed), 2 (attempt was made at constructing a nest), and 3 (nest was made). **(B)**. Representative nests at 7 months. Dual Tg and htau mice constructed the worst nests compared to control mice (*p* < 0.001). 3.5 months: dual Tg: $\bar{X}$ = 1.3816, SD = 0.496, htau: $\bar{X}$ = 1.7826, SD = 0.751, tTA: $\bar{X}$ = 2.5937, SD = 0.471, Wildtype: $\bar{X}$ = 2.6667, SD = 0.604; 7 months: dual Tg: $\bar{X}$ = 1.3947, SD = 0.488, htau: $\bar{X}$ = 1.4783, SD = 0.612, tTA: $\bar{X}$ = 2.3438, SD = 0.729, Wildtype: 2.8214, SD = 0.264.

Two raters blind to experimental conditions rated each nest. Intra-class correlations (ICCs) were calculated between the two raters at both 3.5 and 7 months. There was strong agreement between the two raters' scores of nests at each time point: ICC = 0.899, *p* < 0.001 (3.5 months), ICC = 0.813, *p* < 0.001 (7 months). The two scores were then averaged. Analysis of nesting scores showed a significant main effect of genotype, *F*_(3, 71)_ = 44.682, *p* < 0.001. Dual Tg and htau mice built significantly worse nests than control mice (*p* < 0.001) (Figure 7A), with dual Tg mice consistently having the poorest nests. Examples of representative nests built by each genotype at 7 months can be seen in Figure 7B.

### Brain analysis

Because AD pathology develops with age, brains were analyzed at 8 months of age.

#### Zinpyr-1 (ZP-1) fluorescence

**Figure 8:**
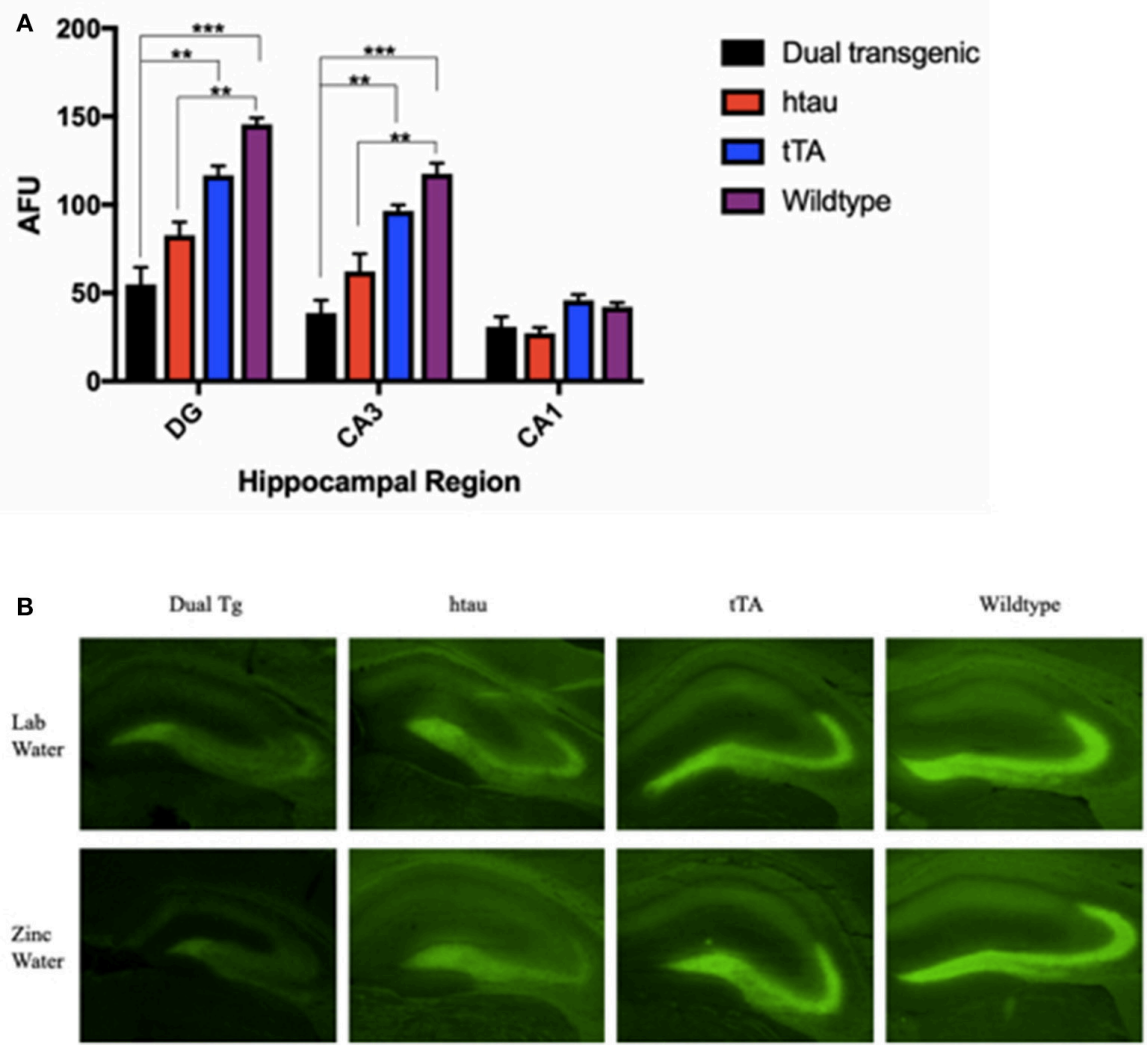
**(A)** Zinpyr-1 fluorescence in the hippocampus. In the dentate gyrus, WT mice (*n* = 6) had significantly more fluorescence (more free Zn^2+^) compared to dual Tg mice (*n* = 7) (*p* < 0.001) and htau mice (*n* = 5) (*p* < 0.01). tTA (*n* = 6) mice also had significantly greater fluorescence than dual Tg mice in the DG (*p* < 0.01). This same pattern was seen in the CA3 region of the hippocampus as well. Bars represent mean ± SEM (**p < 0.01, ***p < 0.001) in arbitrary fluorescence units (AFUs). **(B)** Representative ZP-1 fluorescence. Representative histological sections stained for free Zn using Zinpyr-1.

Fluorescence values (arbitrary fluorescence units—AFUs) from HP regions were analyzed by ZP-1 fluorescence. Hippocampal regions examined included the DG, CA3, and CA1. There was a significant effect of region, *F*_(2, 18)_ = 212.357, *p* < 0.001, and a significant region × genotype interaction, *F*_(6, 18)_ = 18.648, *p* < 0.001 (Figure 8A). In the DG, WT mice had the most fluorescence, indicative of the most free Zn; this was significantly greater than dual Tg mice (*p* < 0.001) and htau mice (*p* < 0.01). tTA mice had significantly greater amounts of fluorescence than dual Tg mice (*p* < 0.01). In the CA3 region, WT mice had significantly more free Zn fluorescence compared to dual Tg (*p* < 0.001) and htau mice (*p* < 0.01). Dual Tg mice had significantly less free Zn than tTA mice (*p* = 0.001). In the CA1 field, there was no significant difference between genotypes.

There was a significant effect of genotype in the HP area examined, *F*_(3, 9)_ = 20.195, *p* < 0.001. Wildtype mice had significantly higher Zn fluorescence values than htau mice (*p* < 0.01). Dual Tg mice had significantly less free Zn than control mice (*p* < 0.01) (Figure 8A). Dual Tg mice had the least Zn across all 3 areas. Histological sections of the HP stained with ZP-1 showing regional differences of Zn fluorescence are shown in Figure 8B.

#### Westerns

##### Inflammation (GFAP)

**Figure 9:**
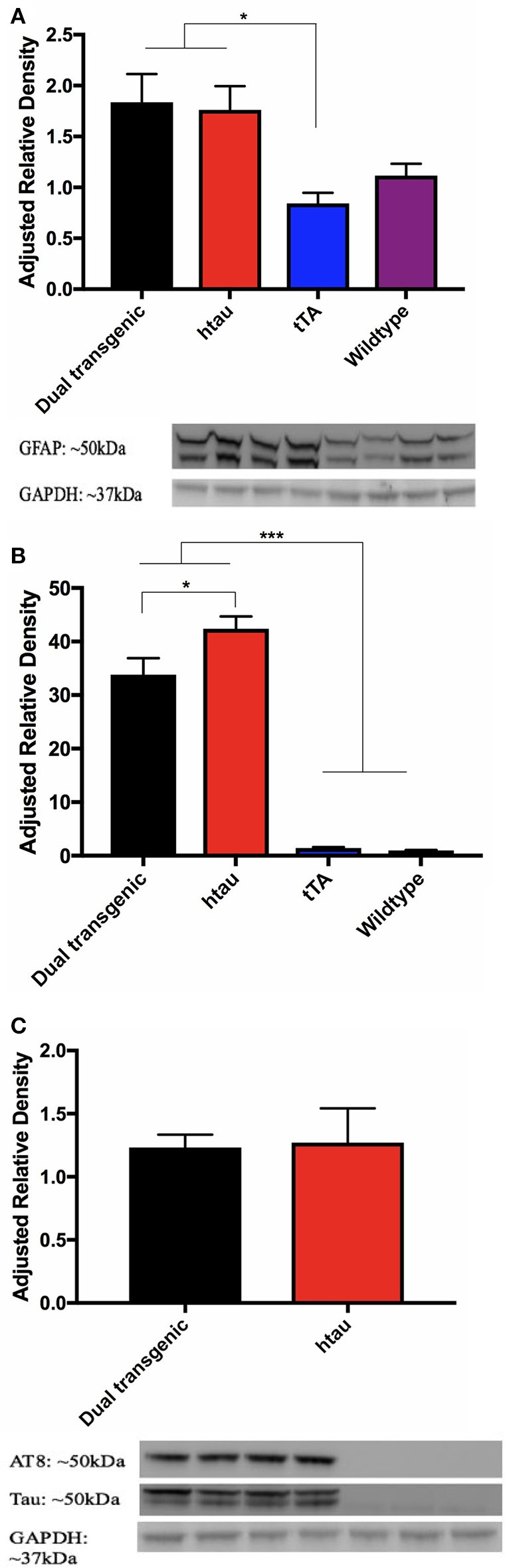
**(A)** GFAP western blots. Dual Tg and htau mice had significantly greater GFAP values than tTA mice (*p < 0.05). Representative western blot shows monoclonal GFAP bands and GAPDH loading control: Dual Tg (Lanes 1–2, *n* = 8), htau (Lanes 3–4, *n* = 8), tTA (Lanes 5–6, *n* = 8), and WT (Lanes 7–8, *n* = 6) dual Tg: $\bar{X}$ = 1.8368, SD = 0.781, htau: $\bar{X}$ = 1.7609, SD = 0.663, tTA: $\bar{X}$ = 0.8437, SD = 0.293, Wildtype: 1.1165, SD = 0.281. **(B)** Total tau western blots. Dual Tg (*n* = 8) and htau mice (*n* = 8) had significantly greater amounts of total than control mice [tTA (*n* = 8), WT (*n* = 6)] (***p < 0.001). htau mice had significantly greater amounts of total tau expression than dual Tg mice (*p < 0.05). **(C)** Phosphorylated tau (AT8) western blots and representative blots. No significant differences were seen between dual Tg (*n* = 8) and htau mice (*n* = 8) in amount of AT8 (phosphorylated tau at sites Ser202/Thr205) detected. Control mice had no detectable signal. Representative western blots show AT8 bands, total tau, and GAPDH loading control. Dual Tg (Lanes 1–2, *n* = 8), htau (Lanes 3–4, *n* = 8), tTA (Lanes 5–6, *n* = 8), and WT (Lane 7, *n* = 6).

Analysis of GFAP band density values (arbitrary units) showed a significant effect of genotype, *F*_(3, 22)_ = 4.992, *p* < 0.01. Dual Tg and htau mice had significantly more GFAP than tTA mice (*p* < 0.05) (Figure 9A). GFAP values showed a negative correlation with nesting scores at 7 months (*r* = −0.352, *p* = 0.056) (Supplementary Figure 7).

##### Tau

Control mice had no detectable bands for AT8; therefore, only dual Tg and htau animals were analyzed for phosphorylated tau (AT8: Ser202/Thr205). A 2 (genotype: dual Tgv. htau) × 2 (water) factorial ANOVA was run on band density values for phosphorylated tau (arbitrary units). A 4 (genotype) × 2 (water) factorial ANOVA was run on band values (arbitrary units) for total tau. For total tau, there was a significant effect of genotype, *F*_(3, 22)_ = 104.216, *p* < 0.001. Dual Tg and htau mice had significantly higher amounts of tau than control mice (*p* < 0.001) (Figure 9B). In addition, htau mice had significantly more tau than dual Tg mice (*p* < 0.05). However, there was no effect of genotype on tau phosphorylation (AT8) expression. There was no effect of water on tau or AT8 protein expression (Figure 9C). Representative blots for tau analysis are presented in Figure 9C.

### Thioflavin fluorescence, tangle pathology

**Figure 10:**
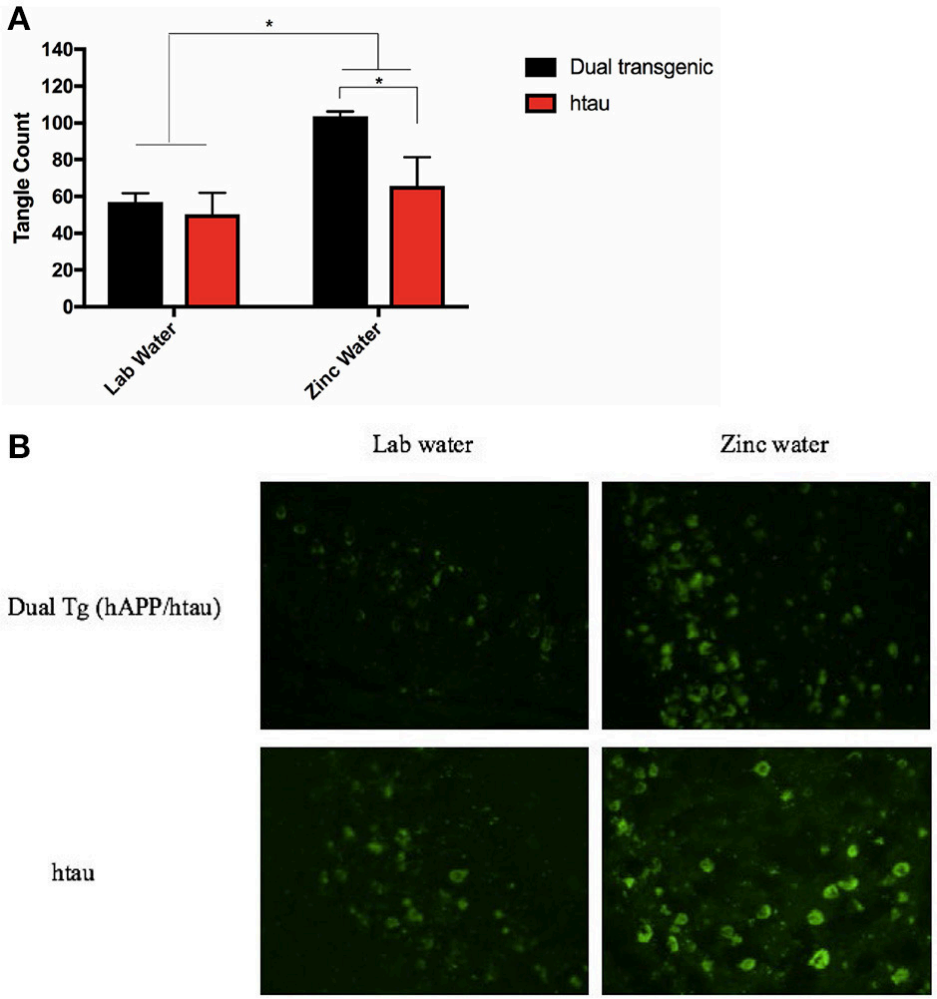
**(A)** Tangle pathology in the HP. Mice given Zn water (*n* = 7) had more tangle pathology detected by Thioflavin-S staining than those given lab water (*n* = 9) (*p < 0.05). Dual Tg mice on Zn water (*n* = 3) had increased tangle pathology compared to htau mice given Zn water (*n* = 4) (*p < 0.05). There was a trending difference (*p* = 0.054) between dual Tg and htau mice where dual Tg mice had more tangles in than did htau mice. Bars represent mean ± SEM. **(B)** Representative tangle pathology. Representative histological sections illustrating Thioflavin-S staining in the HP for dual Tg and htau mice given lab vs. zinc water.

A 2 (genotype: dual Tg v. htau) × 2 (water) factorial ANOVA was run on tangle fluorescence in the HP of Tg mice. There was a significant effect of water, *F*_(1, 12)_ = 8.844, *p* < 0.05, and a trending effect of genotype, *F*_(1, 12)_ = 4.559, *p* = 0.054. Animals on Zn water had significantly more tangle pathology in the HP than those on lab water (*p* < 0.05) and dual Tg animals had more tangle pathology than htau mice. Follow-up analysis revealed that dual Tg mice given Zn water had significantly more tangle pathology than htau mice given Zn water (*p* = 0.032) and dual Tg mice given lab water (*p* < 0.01) (Figure 10A). Representative tangle pathology can be seen in Figure 10B.

### Congo red staining, amyloid pathology

**Figure 11:**
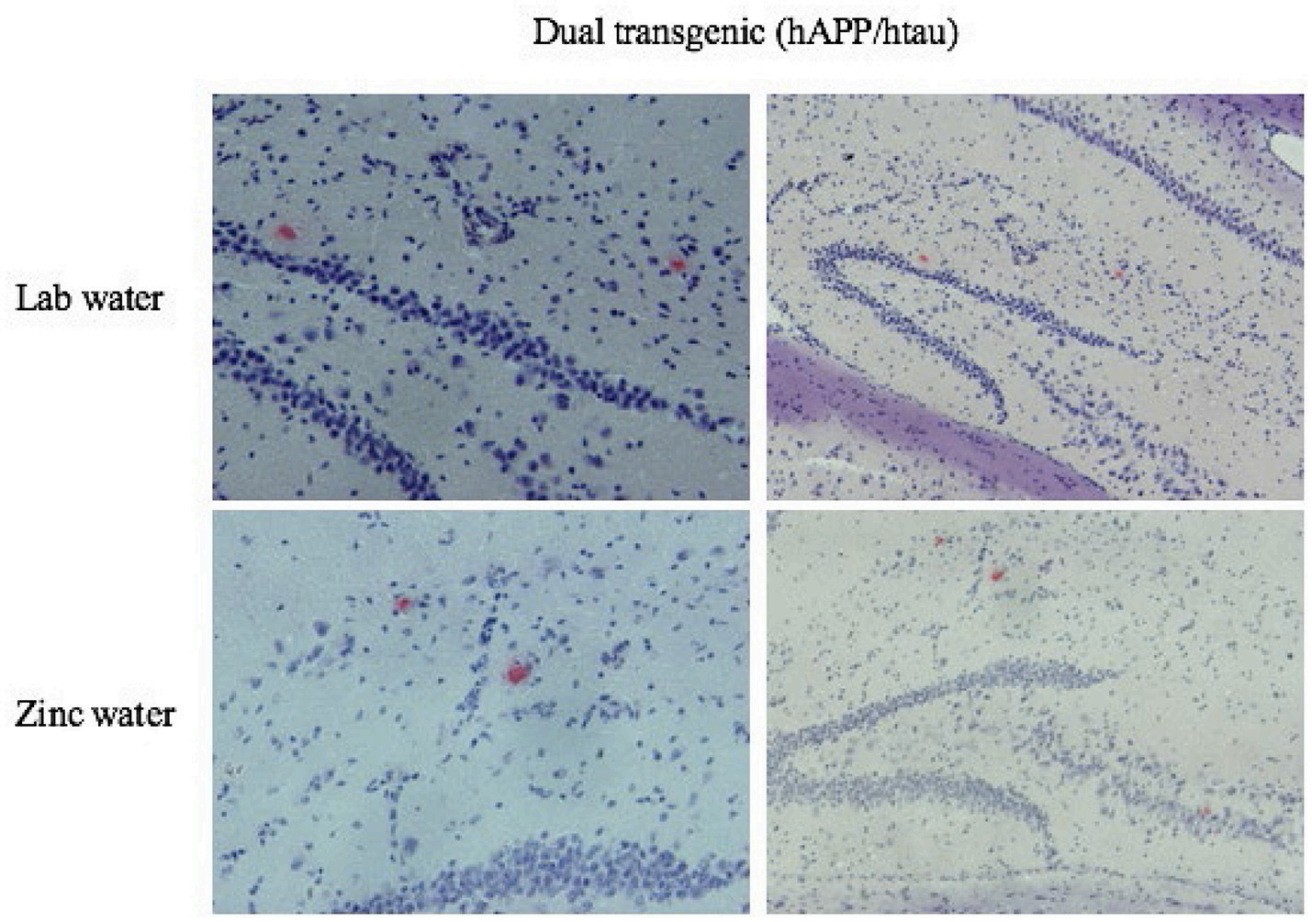
Congo red amyloid plaques. Representative histological sections illustrating Congo Red staining in the HP for dual Tg mice given Zn and lab water at eight months. Images on the left are higher magnifications of the images on the right.

Only dual Tg animals displayed histological plaques (Figure 11). No plaques were observed in the brains of htau or control mice.

## Discussion

This study shows the successful breeding and characterization of a novel dual Tg mouse model that recapitulates many of the deficits seen in AD. This mouse contains both amyloid and tau pathology with no PS1 inclusion, leading to an increased translational potential of this model for future research. There were a number of deficits in both cognitive and non-cognitive behaviors, alongside changes in the brain in this new dual Tg mouse. Overall, as discussed in more detail below, the dual Tg mice showed deficits on all the behavioral measures examined compared to WT mice and either showed them earlier or to a greater extent than htau mice. For example, both the htau and dual Tg mice showed deficits in spatial memory, a hallmark in AD pathology, early on and were increasingly impaired with age, with the dual Tg mice becoming significantly worse than htau mice in BM latency at 7 months (Figure 3B). Dual Tg mice also displayed behavioral disinhibition early on, as seen by their pattern of behavior in the OF and EZM tasks, and they showed significant deficits in non-cognitive daily living measures at an early age, with an inability to build nests at 3.5 months.

In the brain, dual Tg mice have an increased amount of tangle pathology compared to htau mice and display insoluble amyloid plaques in the HP. This plaque pathology at 8 months is consistent with dual Tg mice showing significantly worse spatial memory than htau mice at 7 months. The inclusion of APP causes impairment in HP-dependent behavior compared to those animals with tau alone. Changes in these HP-dependent behaviors were also age dependent, as dual Tg mice were significantly worse than htau mice at 7 months and decreased significantly with age.

Taken together, the behavioral and brain analyses obtained here indicate that mice with both hAPP and tau have significantly greater deficits than those with tau alone. Additional work is needed to determine the relevant importance of the ways in which amyloid and tau can together lead to high levels of toxicity, work which could employ this dual Tg mouse.

While no main effects of Zn administration were noted in behavior, Zn did lead to increased tangle pathology in the HP. The lack of a Zn effect on behavior may be due to the severity of behavioral deficits noted early on in testing; because behavior was already grossly impaired, any potential effects of Zn administration may have been masked.

### Importance of the promoter

An important point to consider in examining behavior, when utilizing mice that need a promoter for genetic expression, is that the presence of the promoter alone should be utilized as a control in addition to a WT group. In order for tau to be expressed in this model, mice must contain both the transgene of interest (Tau P301L) as well as the CaMKIIa promoter under the regulation of the tetracycline-controlled transactivator (tTA) which drives and restricts expression of tau in the forebrain (Mayford et al., 1996; Ramsden et al., 2005). In absence of the tTA-CaMKIIa promoter system, tau pathology will not be noted. The presence of tTA should not be ignored as behavioral and biochemical differences have been noted in these mice compared to WTs (McKinney et al., 2008; Han et al., 2012; Hunsberger et al., 2015). tTA mice have explicitly been used as a suitable control mouse using the rTg4510 mouse (Hunsberger et al., 2015). Mice containing only the promoter show alterations in locomotion compared to WT mice (McKinney et al., 2008) and the background of the mice can impact behavioral changes in mice expressing the promoter (McKinney et al., 2008; Han et al., 2012). Therefore, in the current study, mice containing only tTA were included to control for the promoter system's inclusion.

In the present study, tTA mice differed significantly from dual Tg mice in both cognitive and non-cognitive measures and resembled WT mice; thus, both tTA and WT mice were collectively referred to as control mice. However, at times, tTA mice differed significantly from WT mice and were discussed separately in those instances. At 3.5 months, tTA mice spent significantly less time in the target quadrant of the BM and made more primary errors than WT mice. The fact that tTA and WT mice did not behave the same in each test is an indication that the presence of a promoter system should not be ignored when conducting behavioral tests. Researchers using this mouse should be aware of the presence of the promoter system and should utilize these mice as additional controls in their paradigms. Future work with these promoter mice is warranted to explore mechanisms which may make them deviate from a mouse with no mutations present.

### Behavioral deficits

It is important to assess a breadth of behaviors when characterizing a mouse. In comparison to other experiments with mice carrying amyloid and tau mutations which did not focus on behavior (Lewis et al., 2001; Pérez et al., 2005; Bolmont et al., 2007; Paulson et al., 2008), this mouse was examined on several important behaviors affected in the AD patient: cognitive, emotional, and activities of daily living (ADL) at both early and later stages of disease progression. Although ADL measures are not measured as often as spatial memory deficits, they are easy to perform and provide sensitive measures of deficits. Deficits in the behaviors examined are discussed in more detail below.

### Barnes maze

Hippocampal-dependent tasks are important in Tg animal studies because the HP is affected by AD pathology (Hyman et al., 1984; Braak and Braak, 1991; Serrano-Pozo et al., 2011; Halliday, 2017) and deficits in spatial memory are fairly robust. Both types of Tg mice took longer to find the escape box compared to WT mice at 3.5 and 7 months. At 7 months the dual Tg mice took significantly longer than all other groups (Figure 3B), indicating the severity of their impairment with increased age. Analysis of the 24-h probe trial revealed dual Tg mice again performed significantly worse with age, spending < 25% of the time in the target quadrant at 7 months (Figure 4).

### Zinc and spatial memory

Although no overall Zn effects were seen in the current study, there were several instances where male mice given Zn water displayed spatial memory deficits. This is in agreement with previous literature where in the MWM, male mice given Zn water showed impairments (Linkous et al., 2009). Male mice given Zn took longer to find the escape hole and made more primary errors than those given lab water at 3.5 months. At 7 months, male mice given Zn water spent less time in the target quadrant compared to those given lab water. Zinc water administration has also been seen to lead to C57BL/6J WT mice spending less time in the target quadrant of the BM (Flinn et al., 2014).

### Forced swim

The FS test is considered to be a measure of depressive-type behavior. Transgenic mice swam for longer periods of time (had greater latencies to become immobile) and showed less immobility. The rTg4510 mouse has not been assessed in the FS test previously; however, a study using the P301S tau mouse, found decreased immobility (Takeuchi et al., 2011), consistent with the results shown here.

In the current study, Tg animals spent less time immobile with age, indicative of increased activity. A previous study (Can et al., 2011) provides a possible explanation for this finding. In this study, mice of an FVB/NJ background showed very little time immobile compared to C57BL/6J mice. Considering that the dual Tg mouse is a hybrid background containing FVB/N and C57BL/6J backgrounds, this may explain why there was increased activity. Four dual Tg mice and one htau mouse died before the second testing period and were thus not included in the overall FS analysis. A separate measure of depressive-like behavior, or anhedonia may be better suited for future studies with this new mouse, such as the saccharin-preference test (Willner et al., 1992).

### Locomotion and anxiety: behavioral disinhibition (open field and elevated zero maze)

The dual Tg and htau mice described here exhibit behavioral disinhibition, as observed through behavior in the OF and EZM tasks. Transgenic animals spent less time in the center of the OF and traveled greater distances than control animals, signaling increased anxiety; a result similar to other studies using the OF (Kobayashi and Chen, 2005; Gil-Bea et al., 2007; Takeuchi et al., 2011). Although dual Tg mice traveled significantly greater distances in the OF compared to htau mice early on, by 7 months, there was no difference between dual Tg and htau mice. This initial increase in distance traveled suggests increased anxiety at an earlier time point for the dual Tg mice.

Although this behavior appears to signal anxiety, in the EZM, Tg mice spent more time in the open arms, possibly indicative of less anxiety. Other researchers who have observed a similar result have interpreted this as a lack of inhibition (Lalonde et al., 2003; Gil-Bea et al., 2007). The behavior has been noted in other mouse models of AD where less time in the center of the OF has been accompanied by increased time spent in the open arms of the EZM or elevated plus maze (Takeuchi et al., 2011). In addition, in this study Tg animals made more head dips in the EZM, which involved staying in the open arms peering over the edge. This behavior is thought to be a measure of risk-taking or risk-assessment in rodents (Walf and Frye, 2007); in the current study, this behavior increased over time in the Tg animals.

Head dips made by Tg animals, time spent in the open arms of the EZM, and distance traveled were all higher in Tg animals at both time points tested, indicative of a hyperactive phenotype and disinhibitory tendencies. Human AD patients have been noted to display both increases in anxiety and disinhibition (Mega et al., 1996; Levy et al., 1999) and this may add extra stress on caregivers. In addition, when compared with controls, those with AD have increased disinhibition (Mega et al., 1996).

### Measures of daily living

As AD has important non-cognitive effects as well, measures of daily living are important to assess in this new dual Tg mouse. Alzheimer's patients often show changes in daily living activities that accompany cognitive deficits and place added strain on family members or care takers (Opara, 2012). Alongside changes in ADL measures in AD patients, research has shown that patients with mild cognitive impairment are affected on measures covered under the University of California, San Diego (UCSD) Performance-Based Skills Assessment (UPSA) (Patterson et al., 2001) including planning various activities, household chores, and communication. Severe deficits in these non-cognitive factors are seen early in the disease. Although mice may not balance a check book or dress themselves, non-cognitive tasks should be explored in mouse models of AD. Daily living measures have not been assessed in other mouse models with both amyloid and tau (Table 1A); our mouse shows that these types of behaviors are affected early on and are significantly worse than those seen in control mice.

Dual Tg and htau mice burrowed significantly less pea-gravel than control mice at both time points and showed no improvement of burrowing ability with age. At the earliest point of measurement (3.5 months), there was a very significant difference between Tg and control mice showing that deficits in measures of daily living are seen early.

Nesting behavior has been shown to be impaired in several mouse models of AD, including P301L tau mice (Craven et al., 2018), APPswe/PS1 mice (Filali and Lalonde, 2009), and both CRND8 and CRND8/APOEe4 mice (Graybeal et al., 2015). Dual Tg mice and htau mice built significantly worse nests than controls and showed poor nest construction at 3.5 months. These measures of daily living are severely impaired early in the dual Tg mouse and accompany other deficits in cognition at this early age. It would be interesting to see how early the nesting deficits occur, as this is a measure that can be easily repeated and whether they are accompanied by changes in biochemical measures such as IL-1β and GFAP, which have been seen to correlate with nesting before (Graybeal et al., 2015).

### Zinc in the brain

Loosely-bound or “free” Zn (Zn^2+^) can be detected using fluorescence sensors such as Zinpyr 1 (Woodroofe et al., 2004) and high levels are naturally seen in the hippocampus (Frederickson et al., 1992). Wildtype mice had significantly greater fluorescence than both dual Tg and htau mice in both the DG and the CA3 region of the HP. There were no significant differences between groups in the CA1 region; this region exhibited the lowest values of free Zn. In agreement with previous research (Ketterman and Li, 2008), the hilus of the DG and the CA3 region exhibited the highest levels of fluorescence. Dual Tg mice had the least fluorescence signal in the DG and CA3 region, indicating the least free Zn.

As ZP-1 specifically stains free Zn, it is possible that dual Tg and htau animals had more Zn^2+^ physically bound to misfolded AD proteins, thus making it less available to be bound to ZP-1, and thus detectable via fluorescence. This is supported by previous research that has shown that rTg4510 mice have lower levels of free Zn compared to WT mice (Craven et al., 2018). The dual Tg mouse characterized here has both Aβ plaques and tau tangles. The His residues of Aβ plaques bind Zn^2+^ (Watt et al., 2010; Leal et al., 2012) and key cysteine (Cys) residues in tau can bind Zn^2+^ directly (Mo et al., 2009); this binding to both amyloid and tau can ultimately cause Zn^2+^ to be inaccessible as a free ion.

### Plaque pathology

Dual Tg brains were stained histologically for the presence of Aβ plaques (Congo red). Only dual Tg mice contained plaques in the HP, as expected. There were no significant differences in Congophilic plaque amount between dual Tg mice given lab or Zn water, possibly due to the low number of plaques. Plaque pathology has been shown to be present in J20 mice by 36 weeks (Wright et al., 2013) and mice with the Swedish mutation (K670N/M671L) by 9–10 months (Hsiao et al., 1996). The mice in this study were sacrificed at 8 months of age; this age is relatively early compared to other studies that assess plaque pathology. This dual Tg mouse contained Congo red plaques at 8 months, compared to a hAPP only mouse bearing Swedish and London mutations created by Chabrier et al. (2014), which at 18 months displayed no plaques stained by Congo red. Soluble amyloid could be seen earlier, and amyloid pathology should also be explored biochemically at later stages of the disease in future studies using this mouse.

### Tau phosphorylation(AT8) and tangle pathology

Tau is a phosphoprotein with numerous phosphorylation sites, mainly at Ser and Thr residues (Buée et al., 2000; Gong and Iqbal, 2008; Wang et al., 2013). One antibody of interest was AT8, which is a monoclonal antibody that recognizes tau phosphorylated at Ser202 and Thr205 (Goedert et al., 1995; Otth et al., 2002; Porzig et al., 2007). AT8 has been seen to primarily detect extracellular neurofibrillary tangles (Augustinack et al., 2002). Although no significant differences were seen in AT8 band density between dual Tg and htau mice, htau mice did surprisingly show significantly higher levels of total tau (Figure 9B); this may perhaps be due to the biochemical sample sizes or potential incomplete penetrance (Miko, 2008) of htau when combined with the J20 hAPP mutation.

Dual Tg mice displayed more tangle pathology in the HP than htau mice (*p* = 0.056). Other studies assessing the interaction between Aβ and tau support this increase in tangle pathology in a mouse carrying amyloid and tau, showing that the presence of Aβ can lead to increased NFTs in mice carrying the P301L tau mutation (Götz et al., 2001; Lewis et al., 2001). Amyloid increases tau phosphorylation (Lloret et al., 2011; Nisbet et al., 2015), and both proteins have been seen to alter cognitive behavior compared to non-Tg mice and show increased accumulation of tau compared to htau transgenic mice (Chabrier et al., 2014). We analyzed this dual Tg mouse at 8 months; however, with increased age, this mouse may show an increase in phosphorylated tau, and thus should be explored at later ages.

#### Tangle pathology and zinc

Dual Tg mice displayed more tangle pathology in the HP than htau mice (*p* = 0.056) and dual Tg and htau mice on Zn water had significantly greater tangle pathology than those on lab water (Figure 10A). Dual Tg mice given Zn having the greatest number of tangles. Zn can bind directly to and lead to hyperphosphorylation of tau, which could further impact tau's ability to accumulate (Sun et al., 2012; Huang et al., 2014). Thioflavin-S has been used histochemically to detect beta-pleated sheet secondary structures, a characteristic seen in both Aβ plaques and tangles (Rajamohamedsait and Sigurdsson, 2012). It has also been used in studying neurofibrillary tangle pathology in histochemical sections (Sun et al., 2002; Craven et al., 2018) Comparison of the current histology with work previously done in our lab detecting tangles in the rTg4510 mice (Craven et al., 2018), showed that there were similar histochemical markers. Studies utilizing Thioflavin-S to detect plaque pathology have often revealed bright centers in histochemically marked plaques (Lue et al., 1999; Chishti et al., 2001); in the current study, we did not see this type of reactivity. Plaque pathology, as detected through Congo Red staining and noted above, was minimal for the dual Tg mice. Future studies may wish to utilize other staining methods, such as the Gallyas silver stain to accompany Thioflavin-S fluorescence staining and also examine older mice.

### Inflammation

In AD, glial cells undergo drastic changes that contribute to inflammatory processes. One such glial marker is GFAP, an intermediate filament noted in astrocytes. Dual Tg mice and htau mice displayed significantly more GFAP than tTA mice, possibly indicative of increased reactive astrocytes. Although the level was not significantly different from WT mice, dual Tg and htau mice did have increased mean GFAP values compared to them (Figure 9A). GFAP has been seen to increase with astrocyte activity (Simpson et al., 2010) and to be higher in human AD brains compared to controls (Vehmas et al., 2003; Ingelsson et al., 2004) and astrocytes surround plaques in the AD brain (Vehmas et al., 2003). GFAP, together with IL-1β, was increased in the APP mouse model, CRND8, and has been shown to negatively correlate with activities of daily living measures, (Graybeal et al., 2015). In the current study, there was a trending negative correlation between GFAP values and nesting scores (*r* = −0.352, *p* = 0.056) (Supplementary Figure 7).

#### Impairments in dual Tg and htau transgenic mice

##### Behavior

htau mice showed behavioral disinhibition and deficits in spatial memory along with impairments in burrowing and nesting. Spatial memory deficits were exacerbated in the dual Tg mice and they had the lowest nesting scores. Dual Tg mice showed severe impairments early on in the disease. For example, dual Tg mice spent the least amount of time in the center of the OF test at 3.5 months and in ADL, dual Tg mice initially had lower mean nest values than htau mice and did not improve with age. As the dual Tg mice aged, they diverged from the mice with htau only, highlighting the important role that amyloid's addition can have. From 3.5 to 7 months, dual Tg mice became significantly worse than htau mice in their latency to find an escape hole in the BM and spent significantly less time in the target quadrant, whereas htau mice did not show a significant decrease with age in time spent there. The inclusion of both amyloid and tau in this mouse model could explain why spatial memory became worse with age as pathology worsened.

Behavioral deficits in the Tg mice are robust, given that significant differences persisted despite being housed in cages with running wheels. Previous research has shown that wheel-running exercise in AD mice led to improved performance in various behavioral tasks, including: Barnes Maze (Walker et al., 2015), Y-maze (Maliszewska-Cyna et al., 2016), and the MWM (Adlard et al., 2005). In addition, voluntary wheel running has also been shown to decrease plaque pathology (Adlard et al., 2005).

The dual Tg mouse presented here adds to the literature by exploring a breadth of behaviors, both cognitive and non-cognitive. Other mouse models of AD containing amyloid and tau pathology have focused primarily on brain pathology with limited exploration of behavioral pathology (Lewis et al., 2001; Bolmont et al., 2007; Paulson et al., 2008). Those amyloid/tau models that have assessed behavior have noted similar pathology as seen here; however, we have assessed change over time in several behaviors and included ADL measures. Daily living measures are equally important to measure in animal models of AD and as shown here, these behaviors are impacted early on in disease progression in the dual Tg mouse.

##### Brain analysis

Amyloid plaque pathology was noted in the dual Tg mice at 8 months of age; this is early compared to other mouse models (Chabrier et al., 2014). No plaques were seen in the htau mice, as expected. Similarly, the APP/Tau model explored by Lewis et al. (2001) showed plaques at a relatively early (6 months) with many more noted with age. In tangle pathology, there was a trending difference for greater pathology in the dual mouse compared to htau mice. The inclusion of amyloid may have impacted tau's processing, thus resulting in an increase in tangle pathology and thus an impairment in HP-dependent tasks and learning & memory. This increased tangle pathology was further exacerbated by Zn. An increase in mean inflammation levels was also noted in the dual Tg mouse compared to htau mice. Our data suggest that the inclusion of amyloid in a mouse also containing tau leads to an exacerbation of behavior in several domains, progressive impairments with age, and alterations in brain pathology that can link to deficits seen in behavior. The amyloid cascade hypothesis emphasizes that amyloid is necessary to activate tau (see Nisbet et al., 2015 for review). However, tau may also exacerbate amyloid pathology, as suggested by Ittner and Götz (2011). The interaction between the two is clearly toxic. Therefore, additional studies using mice modeling both amyloid and tau together are of crucial importance. By studying amyloid and tau's interactions *in vivo* and changes in both brain and behavior, we can help unravel how amyloid and tau together lead to pathology seen in Alzheimer's disease.

## Conclusion

In summary, we have characterized a novel dual Tg mouse with mutations in human APP and tau. This was accomplished through a range of behavioral tasks at two time points and the assessment of plaque and tangle pathology. Dual Tg mice showed an increase in tangle pathology compared to htau mice in the HP and performed significantly worse in behavioral tasks. Dual Tg mice had decreased levels of free Zn^2+^, and those on Zn water displayed a significantly higher number of tangles in the HP, perhaps due to Zn's direct binding to the tau protein, suggesting that Zn may be a risk factor for those with AD.

Alzheimer's disease is known as causing impairments in memory, and there is an emphasis on cognitive dysfunction, often measured in mice via the MWM or BM. These dual Tg mice did show significant impairments in the BM both at 3.5 and 7 months; they behaved worse than any other group and their performance deteriorated with age, in contrast to the two control groups whose performance improved. This is in contrast to their performance on non-cognitive tasks, including innate tasks, where a highly significant deficit was observed early on at 3.5 months. Such early deficits, which changed little with age were seen in the open field, a measure of anxiety, the EZM, here considered a measure of disinhibition, and the two ADL activities, burrowing and nesting. Indeed, dual Tg mice were unable to make nests at 3.5 months, the earliest time studied. It is now well known that brain changes in AD begin long before cognitive deficits are seen. These non-cognitive measures, and associated changes in the brain, may provide a better window into AD pathology.

The implications of these results are that experiments should focus on non-cognitive measures as well as cognitive ones, ascertain at what age these deficits are first seen, and look for concurrent brain dysfunction, including measures of inflammation such as IL-1β, GFAP, soluble amyloid, and measures of tau hyperphosphorylation. We hope the use of this mouse will aid in the search for therapeutics to slow or cease the progression of AD, and provide researchers with the ability to gain a more comprehensive understanding of AD.

## Author contributions

SL conducted the behavioral experiments, analyzed data, and wrote the manuscript. MS collected activities of daily living data, served as an animal caretaker throughout the research, and reviewed the manuscript. SL and MS oversaw animal care and completed genotyping and extraction of brain tissue. JF oversaw the project, helped in designing the experiment, and in writing and reviewing the manuscript.

### Conflict of interest statement

The authors declare that the research was conducted in the absence of any commercial or financial relationships that could be construed as a potential conflict of interest. The reviewer AO and handling Editor declared their shared affiliation at the time of the review.

## Supplementary material

Supplementary Figure 1 **(A)** Genotype weights over time. Dual Tg and tau mice weighted significantly less than tTA and WT mice (*p* < 0.05). **(B)** Lab vs. Zinc water weights over time. Mice given lab water weighed significantly more than those given Zn water (*p* < 0.05). **(C)** Male vs. Female weights over time. Male mice weighed significantly more than female mice (*p* < 0.001).

Supplementary Figure 2 Brain size comparisons between genotypes. Dual Tg and htau mice had smaller brains than control mice from general observation. Brains of htau mice had similar brain sizes to the dual Tg mice.

Supplementary Figure 3 **(A)** Latency to enter the center of the OF. Dual Tg and htau mice took longer to enter the center of the OF than control mice (*p* < 0.001). At 7 months, htau mice had longer latencies than tTA (*p* < 0.001) and WT mice (*p* < 0.01) whereas dual Tg mice had longer latencies than just tTA mice (*p* < 0.01). Bars represent mean ± SEM (^**^*p* < 0.01, ^***^*p* < 0.001). **(B)** Total Distance traveled in the OF. With increased age, htau mice traveled significantly greater distances (*p* < 0.05). At 3.5 months, dual Tg mice traveled greater distances than control mice (*p* < 0.001) and htau mice (*p* < 0.01). At 7 months, dual Tg and htau mice traveled greater distances than tTA (*p* = 0.001) and WT mice (*p* < 0.001). Bars represent mean ± SEM (^**^*p* < 0.01, ^***^*p* < 0.001).

Supplementary Figure 4 Differences in Latency to find the escape hole in males on or off Zinc water (3.5 months). Males on lab water found the escape hole significantly faster than those on Zn water (*p* < 0.05) at 3.5 months.

Supplementary Figure 5 **(A)** Percent time spent in the target quadrant of the BM (3.5 months). Dual Tg and htau mice spent significantly less time in the target quadrant at 3.5 months compared to WT mice (*p* < 0.001). Tau mice spent significantly less time in the target quadrant compared to tTA mice (*p* < 0.01) and WT mice spent more time than tTA mice (*p* < 0.05). On days 2 and 3, dual Tg and htau mice spent significantly less time in the target quadrant than WT mice (*p* < 0.01). On day 5, WT mice spent the most time in the target quadrant compared to dual Tg and htau mice (*p* < 0.001). **(B)** Percent time spent in the target quadrant of the BM (7 months). Dual Tg and htau mice spent significantly less time in the target quadrant compared to control mice (*p* < 0.001). On days 2, 3, and 5, dual Tg and htau mice spent significantly less time in the target quadrant than control mice (*p* < 0.001). On day 4, dual Tg spent significantly less time than control mice (*p* < 0.001) and single tau mice spent significantly less time than WT (*p* = 0.001) and tTA mice (*p* < 0.05). **(C)** Differences in the percent of time spent in the target quadrant in male mice on or off Zinc water (7 months). Male mice on Zn water spend less time in the target quadrant than those on lab water (*p* < 0.05) at 7 months.

Supplementary Figure 6 **(A)** Primary errors (3.5 months). At 3.5 months, tTA mice made significantly greater primary errors than WT mice (*p* < 0.01). **(B)** Primary errors (7 months). At 7 months, there was a trending difference between double Tg and WT mice (*p* < 0.10) as well as between htau and WT mice (*p* < 0.10). On day 1, htau mice made significantly more primary errors than tTA mice (*p* < 0.05). **(C)** Primary errors in male mice on or off Zn water (3.5 months). Male mice on Zn water made more primary errors than those on lab water (*p* = 0.05) at 3.5 months.

Supplementary Figure 7 Correlation between inflammation and nesting scores. Nesting score and GFAP adjusted relative density (as assayed through Western Blotting) negatively correlated, *r* = −0.352 (*p* = 0.056). Higher nesting scores (indicative of better nests) were associated with lower GFAP inflammation.
