# Supplementary figures and images for: A Novel hAPP/htau Mouse Model of Alzheimer's Disease: Inclusion of APP With Tau Exacerbates Behavioral Deficits and Zinc Administration Heightens Tangle Pathology
Source: Front Aging Neurosci. 2018 Nov 22;10:382. doi: 10.3389/fnagi.2018.00382 (PMC6263092; doi:10.3389/fnagi.2018.00382)

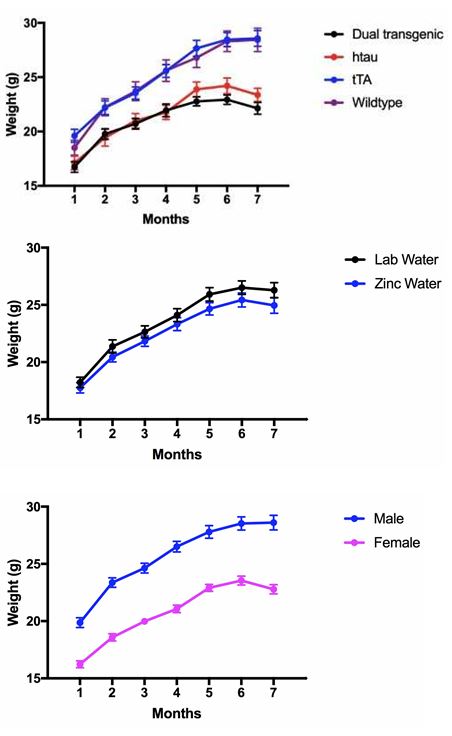

Supplement: Supplementary Figure 1 — (A) Genotype weights over time. Dual Tg and tau mice weighted significantly less than tTA and WT mice (p < 0.05). (B) Lab vs. Zinc water weights over time. Mice given lab water weighed significantly more than those given Zn water (p < 0.05). (C) Male vs. Female weights over time. Male mice weighed significantly more than female mice (p < 0.001). [file Image_1.jpg]

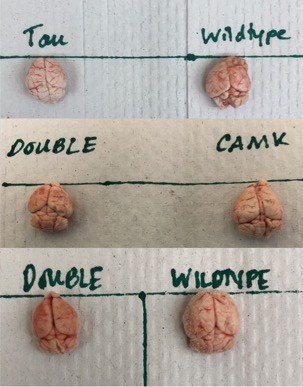

Supplement: Supplementary Figure 2 — Brain size comparisons between genotypes. Dual Tg and htau mice had smaller brains than control mice from general observation. Brains of htau mice had similar brain sizes to the dual Tg mice. [file Image_2.jpeg]

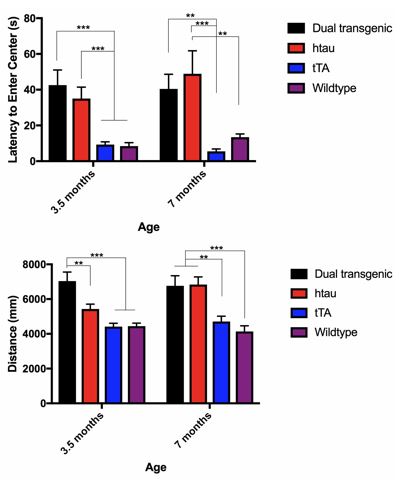

Supplement: Supplementary Figure 3 — (A) Latency to enter the center of the OF. Dual Tg and htau mice took longer to enter the center of the OF than control mice (p < 0.001). At 7 months, htau mice had longer latencies than tTA (p < 0.001) and WT mice (p < 0.01) whereas dual Tg mice had longer latencies than just tTA mice (p < 0.01). Bars represent mean ± SEM (**p < 0.01, ***p < 0.001). (B) Total Distance traveled in the OF. With increased age, htau mice traveled significantly greater distances (p < 0.05). At 3.5 months, dual Tg mice traveled greater distances than control mice (p < 0.001) and htau mice (p < 0.01). At 7 months, dual Tg and htau mice traveled greater distances than tTA (p = 0.001) and WT mice (p < 0.001). Bars represent mean ± SEM (**p < 0.01, ***p < 0.001). [file Image_3.jpg]

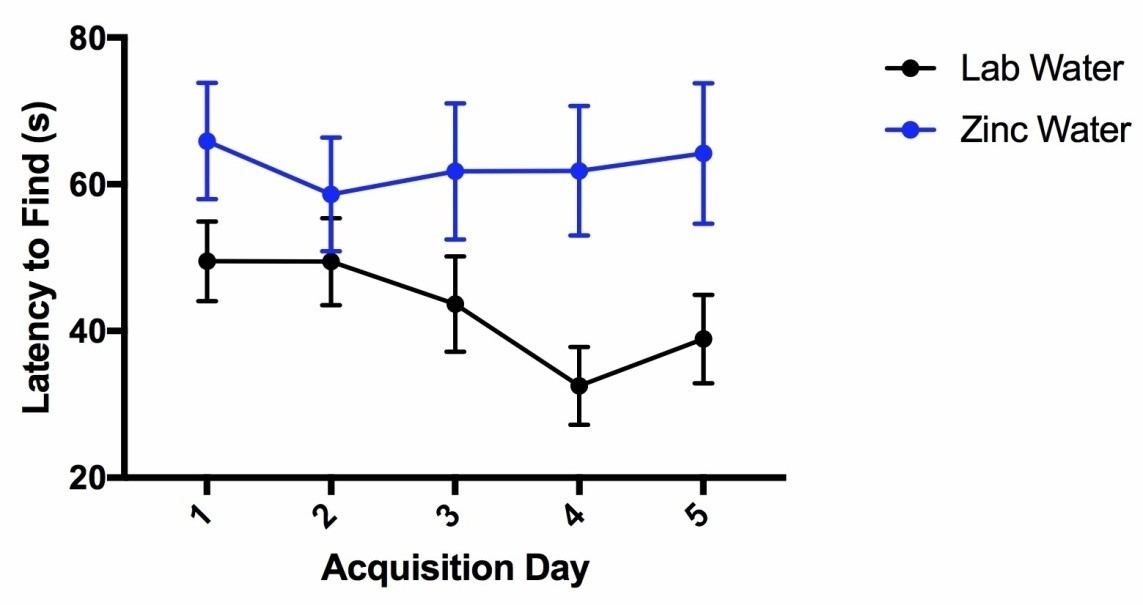

Supplement: Supplementary Figure 4 — Differences in Latency to find the escape hole in males on or off Zinc water (3.5 months). Males on lab water found the escape hole significantly faster than those on Zn water (p < 0.05) at 3.5 months. [file Image_4.jpeg]

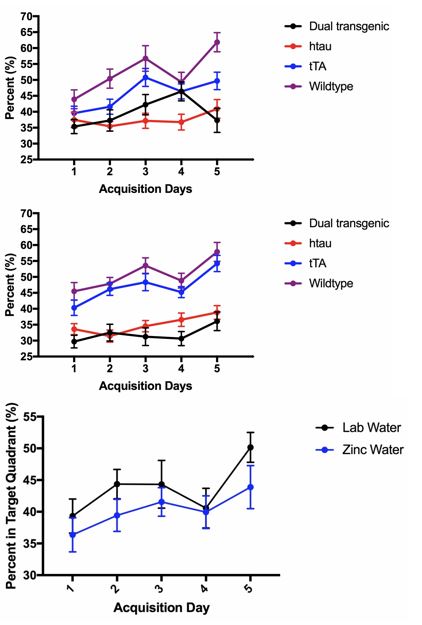

Supplement: Supplementary Figure 5 — (A) Percent time spent in the target quadrant of the BM (3.5 months). Dual Tg and htau mice spent significantly less time in the target quadrant at 3.5 months compared to WT mice (p < 0.001). Tau mice spent significantly less time in the target quadrant compared to tTA mice (p < 0.01) and WT mice spent more time than tTA mice (p < 0.05). On days 2 and 3, dual Tg and htau mice spent significantly less time in the target quadrant than WT mice (p < 0.01). On day 5, WT mice spent the most time in the target quadrant compared to dual Tg and htau mice (p < 0.001). (B) Percent time spent in the target quadrant of the BM (7 months). Dual Tg and htau mice spent significantly less time in the target quadrant compared to control mice (p < 0.001). On days 2, 3, and 5, dual Tg and htau mice spent significantly less time in the target quadrant than control mice (p < 0.001). On day 4, dual Tg spent significantly less time than control mice (p < 0.001) and single tau mice spent significantly less time than WT (p = 0.001) and tTA mice (p < 0.05). (C) Differences in the percent of time spent in the target quadrant in male mice on or off Zinc water (7 months). Male mice on Zn water spend less time in the target quadrant than those on lab water (p < 0.05) at 7 months. [file Image_5.jpg]

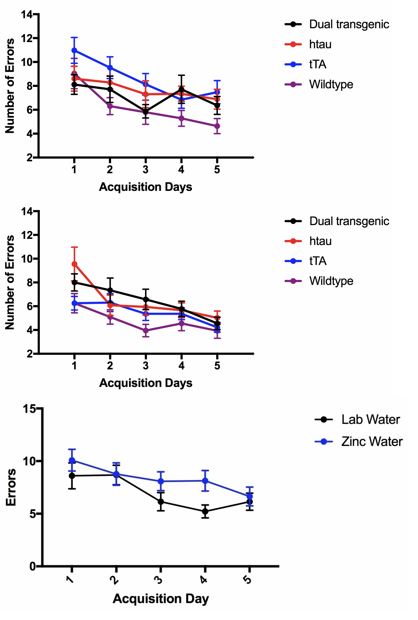

Supplement: Supplementary Figure 6 — (A) Primary errors (3.5 months). At 3.5 months, tTA mice made significantly greater primary errors than WT mice (p < 0.01). (B) Primary errors (7 months). At 7 months, there was a trending difference between double Tg and WT mice (p < 0.10) as well as between htau and WT mice (p < 0.10). On day 1, htau mice made significantly more primary errors than tTA mice (p < 0.05). (C) Primary errors in male mice on or off Zn water (3.5 months). Male mice on Zn water made more primary errors than those on lab water (p = 0.05) at 3.5 months. [file Image_6.jpg]

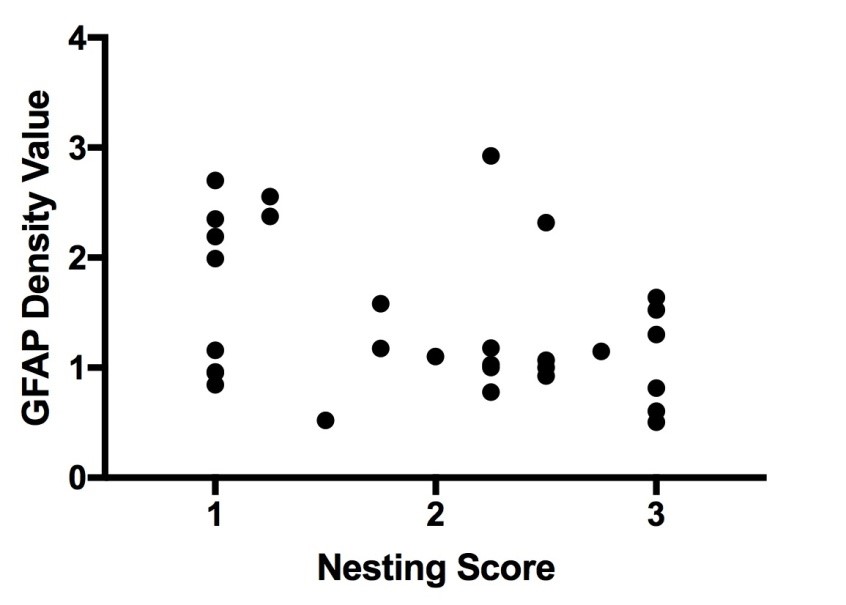

Supplement: Supplementary Figure 7 — Correlation between inflammation and nesting scores. Nesting score and GFAP adjusted relative density (as assayed through Western Blotting) negatively correlated, r = −0.352 (p = 0.056). Higher nesting scores (indicative of better nests) were associated with lower GFAP inflammation. [file Image_7.jpeg]
